# The miRNome of Depression

**DOI:** 10.3390/ijms222111312

**Published:** 2021-10-20

**Authors:** Dariusz Żurawek, Gustavo Turecki

**Affiliations:** 1 McGill Group for Suicide Studies, Department of Psychiatry, Douglas Mental Health University Institute, McGill University, 6875 LaSalle Blvd, Montreal, QC H4H 1R3, Canada; 2 Department of General Biochemistry, Faculty of Biochemistry, Biophysics, and Biotechnology, Jagiellonian University, Gronostajowa 7 Street, 30-387 Kraków, Poland; dariusz.zurawek@uj.edu.pl

**Keywords:** microRNA, depression, antidepressant treatment, human brain, biomarker, miRNome

## Abstract

Depression is an effect of complex interactions between genetic, epigenetic and environmental factors. It is well established that stress responses are associated with multiple modest and often dynamic molecular changes in the homeostatic balance, rather than with a single genetic factor that has a strong phenotypic penetration. As depression is a multifaceted phenotype, it is important to study biochemical pathways that can regulate the overall allostasis of the brain. One such biological system that has the potential to fine-tune a multitude of diverse molecular processes is RNA interference (RNAi). RNAi is an epigenetic process showing a very low level of evolutionary diversity, and relies on the posttranscriptional regulation of gene expression using, in the case of mammals, primarily short (17–23 nucleotides) noncoding RNA transcripts called microRNAs (miRNA). In this review, our objective was to examine, summarize and discuss recent advances in the field of biomedical and clinical research on the role of miRNA-mediated regulation of gene expression in the development of depression. We focused on studies investigating post-mortem brain tissue of individuals with depression, as well as research aiming to elucidate the biomarker potential of miRNAs in depression and antidepressant response.

## 1. Introduction

Prolonged stressful conditions, including traumatic events, act as risk factors for depressive psychopathology. Accordingly, it is well established that stress experienced in the context of adversity is a common trigger for depressive episodes (major depressive disorder-MDD). Depression is associated with abnormalities in the maintenance of homeostasis and normal neuronal activity in many regions of the brain [1]. These abnormalities condition the reduced biological ability of an individual to actively cope with stressful factors. During the past few decades, many interesting basic and clinical studies have been carried out to elucidate the molecular and physiological determinants of depression. The serotoninergic, noradrenergic and dopaminergic neurotransmitter systems have been at center stage both in the investigation of the neurobiological bases of depression, as well as in the development of therapeutic strategies [2,3,4,5]. Disturbed transmission of the above-mentioned neurotransmitters is observed in people suffering from depression [6] as well as in animal models of depression [7]. Currently, antidepressant therapies are based on the use of drugs targeting the main monoaminergic systems (including reuptake inhibition or autoreceptor antagonism). The effects of antidepressant therapy with drugs that regulate serotonergic or noradrenergic transmission occur only after several weeks of use, while drugs that affect the dopaminergic system, due to the possibility of addiction, are used marginally [8]. Although drugs which modulate serotonergic and/or noradrenergic neurotransmitter systems show effective antidepressant action [9], a significant proportion of individuals with depressive disorders do not respond to a given antidepressant treatment [10,11]. This clearly indicates that the pathophysiological mechanisms underlying depression may be more complex and occur not only at the level of monoaminergic neuromodulatory signaling in the brain.

Recent studies suggest that stress-related mood disorders are an effect of complex genetic, epigenetic and environmental interactions [12,13,14,15]. Stress responses are more associated with multiple small and often dynamic molecular changes in homeostatic balance, rather than with one genetic factor of strong phenotypic penetration [16,17,18,19]. As depression is a multifaceted phenotype, it is important to study the biochemical pathways that can regulate overall allostasis of the brain. One such biological system that has the potential to fine-tune different biological pathways is RNA interference (RNAi). RNAi is an epigenetic process showing a very low level of evolutionary diversity and relies on the posttranscriptional regulation of gene expression using, in the case of mammals, primarily short (17–23 nucleotides) and noncoding RNA transcripts called microRNAs (miRNA). 

The elucidation of the role of RNA-interference in animals and the classification of miRNAs as a new group of posttranscriptional regulators of gene expression took place in the 1990s. In 1993, Lee and co-workers reported that in the nematode C. elegans, the lin-4 gene encodes the short and noncoding RNA molecules which are responsible for time-dependent repression of the LIN-14 protein during larval development [20]. Several years later, in 1998, Fire and co-workers observed a potent gene silencing effect in C. elegans injected with double stranded RNA molecules targeting the ucn-22 gene [21]. 

At present, approximately 2000 distinct miRNAs have been annotated in the human genome (miRbase v22 database) [22,23]. The majority of these are expressed in the brain and some of them, such as e.g., miR-9-5p, miR-124-3p, are even considered as brain-specific [24,25]. These observations led to the hypothesis that miRNAs may be important factors controlling the homeostatic balance in the central nervous system, and their regulatory role may be disturbed by stress and trauma. Currently, the involvement of mature miRNAs in the pathophysiology of depression and other neuropsychiatric disorders is under intense investigation. Recent research has shown that susceptibility to stress may correlate with changes in the level of miRNA expression. This was observed in many different brain regions of stressed animals in models of depression, as well as in post-mortem human brain studies of individuals affected with major depression.

In this review, our objective was to examine, summarize and discuss recent advances in the field of biomedical and clinical research on the role of miRNA-mediated regulation of gene expression in the development of depression. We focused on studies investigating the post-mortem brain tissue of individuals with depression, as well as research aiming to elucidate the biomarker potential of miRNAs in depression and antidepressant response. The review is organized in five main sections, of which the first is an introduction to the topic. In the second chapter we describe the biogenesis pathway and biochemical properties of mature miRNAs that are important for miRNA-mediated gene fine-tuning and in biomarker studies. In the third chapter we discuss studies investigating miRNAs in human brain tissue samples collected from depressed individuals who died by suicide. The fourth chapter is focused on clinical studies investigating miRNAs in different peripheral tissues and body fluids in depressed patients, as well as the effects of antidepressant treatments. The last section summarizes current results and is particularly focused on the specific miRNAs that consistently appeared across independent studies using brain tissue, body fluids, and antidepressant treatment.

## 2. miRNA Biogenesis, Function and Regulatory Properties

miRNAs are short (17–23 nucleotides), noncoding, single-stranded RNA transcripts that, in mammals, are responsible for the sequence-specific and post-transcriptional regulation of expression of more than 60% of genes [26]. miRNAs are endogenously encoded in the mammalian genome and are initially transcribed by polymerase II (Pol II) as long (up to several hundreds of nucleotides) primary transcripts (pri-miRNAs). Pri-miRNAs are then trimmed in the nucleus into shorter (~65–70 nt) stem-loop secondary structures named precursor miRNAs (pre-miRNAs). The microprocessor complex consisting of two proteins, DiGeorge Syndrome Critical Region 8 (DGCR8) and Drosha, is responsible for the enzymatic processing of pri-miRNAs to pre-miRNAs in the nucleus. DGCR8 is a protein which recognizes stem-loop structures in pri-miRNA. Drosha is a Class 2 RNase III enzyme that cuts off pre-miRNA hairpins from primary transcripts. A single pri-miRNA may contain up to six miRNA precursors. After processing in the nucleus, pre-miRNA transcripts are transported to the cytoplasm via a transporter-Exportin-5 (XPO5), which is a member of the karyopherin family of nucleocytoplasmic transport factors. The exported pre-miRNAs are further processed in the cytoplasm by the enzyme Dicer into approximately 22 nucleotides-long RNA duplexes. Dicer, which belongs to the RNase III family, cleaves double-stranded RNA molecules and, together with the enzyme Drosha, plays a crucial role in the mature miRNA biogenesis system [27]. After Dicer processing, small RNA duplexes are incorporated into a protein complex named RISC (RNA-induced gene silencing complex). In RISC, one strand of the duplex is removed and the other strand (17–23 nt) constitutes mature miRNA. A core component of RISC is protein Argonaute 2 protein (AGO2). It belongs to an evolutionarily conserved protein family with intrinsic endonuclease activity, which is responsible for the final step of miRNA maturation [28]. RISC containing AGO2 and a guide strand of mature miRNA is a multiple-turnover enzymatic complex responsible for nucleolytic destruction or inhibition of the translation of mRNA transcripts. Therefore, RISC may directly regulate the protein levels of a given mRNA. It has been widely observed that the specificity of the action of the RISC complex results from perfect Watson-Crick pairing between six nucleotides at positions 2–7 at the 5′- end of the mature miRNA, named the seed region, and a complementary sequence located mainly in the 3′ untranslated region (UTR) region of target mRNAs. This interaction is even more efficient and thus biologically promoted when additional pairing of nucleotides at positions one and eight, flanking the miRNA seed region, takes place [29,30]. It is not difficult to imagine that since the RISC specificity depends on the complementarity of only eight nucleotides between a mature miRNA and its target, one miRNA transcript can negatively regulate the translation of multiple different mRNAs. Accordingly, the alteration of the levels of a single miRNA may lead to multiplicative downstream effects that may change entire biochemical pathways [29,31]. The pleiotropic effects of miRNAs on the regulation of gene expression seems to be an important factor in maintaining the homeostatic balance of the brain in response to stress. This is why miRNA-mediated gene regulation is currently under intensive investigation in many basic and clinical studies which are trying to delineate the mechanisms underlying stress susceptibility and resilience. miRNAs possess another feature which may be important in the regulation of a homeostatic balance in the brain. One mRNA transcript may contain multiple sites complementary to many unrelated miRNAs, and thus whole nets of different miRNAs may converge their action on the same gene or functionally related pathways [32]. In fact, this phenomenon was reported to be important for normal mammalian brain development [33] and functioning [34,35]. A third key feature of miRNAs is their very high level of evolutionary conservation among mammals, which not only suggests their importance for physiology but also makes them ideal candidates for translational studies [36].

## 3. Studies Investigating Depression-Related miRNAs in the Brain

### 3.1. Brodmann Area 9 (BA9) 

BA9 is a region of the human frontal cortex that it is involved in working memory [37,38], memory-guided attention [39], as well as the processing of emotions [40]. Abnormalities in biochemical functioning (including miRNA regulation) of the BA9 region have been observed in patients suffering from bipolar disorder [41] and depression [42]. For instance, Smalheiser and co-workers reported altered expression levels of 21 different miRNAs in BA9 of depressed patients [43]. Interestingly, many of the dysregulated miRNAs were located in close proximity in the genome, and shared similarities in seed regions, and thus shared putative mRNA targets [43]. A more recent study reported increased expression levels of miR-30a-5p and miR-30e-5p in BA9 of individuals with depression who died by suicide [44]. miR-30a-5p and miR-30e-5p negatively regulated the expression level of ZDHHC21, a gene responsible for the palmitoylation of the serotonin autoreceptor 1A (HTR1A). Diminished palmitoylation of HTR1A receptor, resulting in disturbed signaling properties, was observed by the authors, not only in brain tissue of depressed suicides but also in the prefrontal cortex of anhedonic mice in a chronic stress paradigm [44]. miRNAs expressed in the BA9 area may also be a molecular link between dementia and late-life depression in humans [45]. Wingo et al. performed a longitudinal analysis of the development of depressive symptoms and dementia among 300 elderly participants. After their death, they examined global miRNA alterations in BA9 and found that changes in the expression levels of four miRNAs i.e., miR-484-5p, miR-26b-5p, miR-30d-5p and miR-197-3p, were significantly associated with late-life depressive symptoms. Subsequent analysis on a replication cohort showed consistent changes for miR-484-5p. Lower levels of miR-484 expression in the BA9 region accompanied higher symptoms of late-life depression and a higher probability of having Alzheimer’s dementia [45]. 

### 3.2. Brodmann Area 10 (BA10)

The BA10 region is part of the anterior prefrontal cortex [46] and is highly developed in humans. The volume of BA10 in humans is significantly larger than corresponding regions observed in other primates [47]. BA10 is involved in higher cognitive processing, including future-thinking [48] or mediation of reward related behavior [49]. Interestingly, both functions have been observed to be disturbed in depressed subjects [48,49,50]. The first study, made by our group, reported upregulation of miR-185* and miR-491-3p in the BA10 region of individuals who died by suicide [51]. miR-185* was responsible for the negative regulation of the expression level of TRK2-1 receptor and thus, may contribute to diminished BDNF-related signaling in suicide completers [51]. Another study reported decreased expression levels of miR-508-3p and miR-152-3p in the BA10 region of individuals who died by suicide as compared to non-depressed controls [52]. Additionally, it has been observed that individuals who died by suicide had upregulated expression of miR-19a-3p in BA10 regardless of their previous psychiatric diagnosis. This was, however, not observed in brains of MDD patients who died by causes other than suicide [53]. Further examination of miR-19a-3p target genes suggested that it may be involved in the modulation of TNFa signaling [53] and genes responsible for regulation of neuroinflammation [54]. 

### 3.3. Brodmann Area 44 (BA44)

BA44 in the human brain is located in the third frontal convolution of the frontal lobe [55]. BA44—especially that from the left hemisphere—is functionally engaged in different aspects of speech and higher cognitive functions, such as learning, imitation, creation of music [56], and expression of emotional information [57]. BA44 is also another well-studied cortical region in terms of changes in the levels of miRNA expression in depression and suicide. Significant upregulation of the expression of miR-34c-5p, miR-139-5p, miR-195-5p, and miR-320c has been observed in the BA44 area of depressed suicide completers [58]. Current research using animal models of stress also supported the observation that upregulation of miR-34c-5p and miR-139-5p in the brain may be associated with the development of stress-related mood disorders. In rodents, acute stress induced an increased expression of miR-34c-5p in the amygdala [59]. Chronic stress, in turn, was associated with the upregulation of miR-34c-5p in the nucleus accumbens [60] and the amygdala [59], while experiencing early life stress caused the aforementioned changes in the hippocampus, prefrontal cortex, and amygdala [61]. It has been observed that blood-derived exosomes collected from MDD patients had the potential to evoke depressive-like symptoms in mice [62]. This effect was hypothesized to be mediated by miR-139-5p, whose levels in blood-derived exosomes were significantly higher in MDD patients than in healthy controls [62]. Furthermore, mice that experienced chronic unpredictable stress had higher levels of miR-139-5p expression in the brain, while intranasal administration of a miR-139-5p antagomir alleviated the depressive-like symptoms in stressed animals [62]. The expression level of miR-1202 was significantly downregulated in BA44 of depressed patients who died by suicide compared to healthy persons [63]. Interestingly, miR-1202 is primate-specific and its expression is enriched in the brain [63]. It has been observed that the expression level of miR-1202 is significantly higher in suicides that were treated with antidepressants as compared to those with no treatment history, but still significantly lower than in healthy controls [63]. This may indicate that miR-1202 is involved in the pathophysiological mechanisms of depression, suicidal behavior, and can be a potential biomarker of the response to antidepressant treatment. In another study, Torres-Berrio et al. demonstrated that miR-218 is a posttranscriptional repressor of netrin-1 guidance cue receptor (DCC) and its reduced level, together with the exaggerated expression of DCC in the BA44 cortical region of depressed individuals, may contribute to the stress-vulnerable phenotype [64]. In addition, the authors observed consistent changes in the prefrontal cortex of mice susceptible to chronic stress. DCC is responsible for controlling synapse formation, axon arborization, and the organization of neural connectivity. Therefore, miR-218-dependent regulation of the DCC level in the brain may be a target of future antidepressant therapeutic interventions [64]. Based on exploratory bioinformatic analysis of five microarray datasets investigating miRNA expression in BA44, Wang and co-workers concluded that miR-124-3p is one of the most dysregulated miRNAs in this region in MDD [65]. They observed a general decrease in the level of miR-124-3p in BA44, particularly in male patients diagnosed with MDD [65]. 

### 3.4. Anterior Cingulate Cortex (ACC)

The ACC is a cortical structure localized in the medial wall of each hemisphere in the ventromedial frontal cortex [66,67] and was originally divided by Brodmann to subareas BA24, BA25, BA32, and BA33 [66]. The ACC is a brain structure of particular interest to depression because of its unique function and connectivity. The ACC has strong neuroanatomical connections with limbic structures, which regulate reward-related behaviors, and the prefrontal cortex, which is responsible for cognition [66]. Thus, the ACC is considered as a region integrating functional outputs from both limbic and prefrontal cortical circuits. The ACC is thought to be implicated in the regulation of positive and negative emotional feelings, mood [68,69], attention and salience [69,70]. It also appears to be affected in depressed patients [71,72], and its functional connectivity with the ventromedial prefrontal cortex correlates with antidepressant treatment response [73]. By using next generation sequencing (NGS), Yoshino et al. observed the upregulation of 117 and the downregulation of 54 different miRNAs in the ACC of depressed subjects [74]. This accounted for 4.16% and 2.13%, respectively, of the total number of miRNAs examined in this study [74]. These results may suggest that the altered miRNA landscape in the ACC may be involved in the development of depression. In another study by our group, Fiori et al. used NGS in two different human brain regions, i.e., BA24 (which is part of ACC) and the lateral habenula, and reported similar expression patterns of 32 miRNAs between both brain regions, of which miR-204-5p, miR-320b, miR-323a-3p and miR-331-3p showed increased expression in depressed patients [75]. Further experiments also indicated that miR-323a-3p may be involved in the development of the depressive phenotype by the regulation of neuregulin signaling in both brain structures [75]. This observation is also supported by preclinical experiments in which prenatal stress upregulated miR-323a-3p in the brain of a rat [76]. Azevedo and co-workers also focused on the BA24 subregion of ACC. They found decreased expression of miR-34a, miR-184, and miR-195, however, none of them passed correction for multiple testing [77].

### 3.5. Other Brain Regions

So far, most studies investigating the role of miRNAs in pathophysiological mechanisms of depression in the human brain have focused on cortical regions such as ACC, BA44 or the dorso-lateral prefrontal cortex (for more details, see Table 1). However, there is only limited information on changes in miRNA expression in other areas of the brain which are also affected by stress. For instance, Roy and colleagues examined miRNA networks in the locus coeruleus (LC), which is a brain region where norepinephrinergic neurons are located, in depressed suicides and healthy controls. They found differential regulation of 13 miRNAs (see Table 1) and by using functional clustering of predicted target genes, they showed that this group of miRNAs may be responsible for the dysregulation of cellular biological pathways involved in normal brain functioning [78]. The depressive phenotype was also related to the miR-511-mediated reduction of GDNF family receptor alpha 1 (GFRA1) signaling [79] and miR-128-3p-mediated downregulation of key target genes of the Wnt signaling pathway in basolateral amygdala [80]. miR-511 has also been reported as a miRNA that may play a significant role in the regulation of brain responses to stress in animals. miR-511 has been found to bind to FKBP5 mRNA, the chaperone gene for the glucocorticoid receptor. This gene is significantly involved in stress responses and regulates glucocorticoid signaling in the brain [81,82]. In animal studies, reduced miR-511 expression was observed in the prefrontal cortex in rats, following chronic unpredictable stress or dexamethasone administration [82]. In a mouse model of post-traumatic stress disorder (PTSD), lower miR-511 transcript levels were observed in the hippocampus and hypothalamus of stress-susceptible animals, while in the medial prefrontal cortex both stress-susceptible and resilient animals had lower levels of miR-511 [83]. Another interesting study was performed by Issler et al. [84]. In this study, the authors used a series of complementary animal and human approaches, and observed that miR-135a-5p levels were decreased in the dorsal raphe (RN) and raphe magnus (RM), areas containing primarily projecting serotonergic neurons, which provide a substantial amount of serotonergic innervation to the forebrain. It is worth noting that both the serotonin transporter (SERT) and the serotonin autoreceptor (HTR1A) were validated as targets regulated by miR-135a-5p. The authors extended their research on animal studies and observed that although chronic social defeat stress did not alter miR-135a-5p in the mouse RN, chronic and acute administration of imipramine and fluoxetine increased expression levels of miR-135a-5p in the RN of both stressed and non-stressed animals [84]. Furthermore, animals with miR-135a-5p overexpression in the RN demonstrated resilience to the adverse effects of chronic stress, while its knockdown promoted anxiety-like behavior and attenuated the response to antidepressants [84]. Taken together, these results suggest that miR-135a-5p may play an important role as an endogenous factor that regulates antidepressant behavior [84].

## 4. miRNA Studies of Depression and Antidepressant Treatments Investigating Blood and Body

Identifying noninvasive peripheral biomarkers that may reflect pathophysiologic changes occurring in the depressed brain is of prime importance in the clinic. The discovery of such a biomarker which, in addition may also predict antidepressant response, could overcome major obstacles in clinical practice, including the individual and subjective evaluation of symptoms of depression, and the trial-and-error processes currently used in the selection of the most effective antidepressant therapy. In this context, miRNAs found in blood and body fluids are currently gaining attention [88]. Circulating miRNAs appear to be good candidates in biomarker research due to their high level of stability in the blood [89,90] and the presence of brain-specific miRNA transcripts, such as miR-9, that can be detected in body fluids [91]. Indeed, numerous scientific reports have shown that brain-specific miRNAs can effectively cross the blood–brain barrier and are detectable in blood [85,86,87,88,89,90,91,92], as well as in brain-derived extracellular vesicles circulating in the bloodstream [93]. The ability of brain-specific miRNAs to pass through the blood–brain barrier makes them ideal biomarkers of brain processes, including mental illnesses such as depression. 

Undoubtedly, blood is the most commonly used biological material in clinical studies, as it is relatively easy to obtain and does not require special processing. Nevertheless, it should be taken into account that whole blood contains a high proportion of erythrocytes and other cells, which are a vital source of miRNAs not directly related to functioning of the brain. Thus, discrete changes, which may be related to brain-specific processes and potentially present in the blood, may not be detected as they could be “diluted” among other signals, especially when the same miRNA is expressed in both blood and brain cells. To overcome this potential obstacle, some authors focused on the examination of plasma and serum samples. Others went a step further and used cerebrospinal fluid (CSF) and, more recently, investigated extracellular vesicles circulating in the blood from other tissue origins, including the brain. Examining extracellular vesicles is an exciting avenue that may maximize the sensitivity and specificity of brain-derived miRNA changes that can be detected in the bloodstream.

### 4.1. Whole Blood

Using whole blood, one study found that twelve weeks of escitalopram treatment increased the expression of 28 miRNAs and decreased miR-34c-5p and miR-770-5p expression in the blood of depressed patients [94]. Another study found that 4 weeks of citalopram treatment normalized blood levels of six downregulated and four upregulated miRNAs in depressed subjects [95]. For further details, see Table 2. Interestingly, both antidepressant treatments resulted in the upregulation of the level of miR-335 [94,95]. miR-335 has the potential to interact with glutamate metabotropic receptor 4 (GRM4) which has been implicated in the control of anxiety-related behavior [95]. Our group has also observed that depressed patients had decreased blood levels of miR-1202 while 8 weeks of escitalopram [63] or desvenlafaxine [96] treatment were able to normalize these changes. The blood level of miR-1202 has also been shown to be a promising marker of antidepressant response efficacy in depressed patients who received 8-week duloxetine (serotonin-noradrenaline reuptake inhibitor–SNRI) treatment [97]. Of interest, similar to miR-355, miR-1202 regulates GRM4 signaling and thus contributes to the control of stress-related behaviors [63]. Decreased blood levels of miR-135a-5p were reported in depressed patients [84,98] and cognitive-behavioral therapy was able to reverse these changes [84]. Furthermore, overexpression of miR-135a-5p in the mouse midbrain resulted in a stress-resilient phenotype [84], while chronic treatment of stressed mice by a miR-135a-5p mimic had antidepressant effects [98]. In another study, Fiori et al. examined, among other miRNAs, whole blood levels of miR-16-5p in two independent cohorts of depressed patients treated with duloxetine. During the study they examined two groups of patients who showed different responses to given therapies, and observed a strong tendency, especially in responders, toward a lower blood level of miR-16-5p [97], which is partially consistent with previous reports. Interestingly, duloxetine did not appear to affect blood miR-16-5p levels. Recent scientific observations suggest that miR-16-5p mediates the molecular action of selective-serotonin reuptake inhibitors (SSRIs) [99] but not SNRI [100], which may not only explain the lack of miR-16-5p changes in duloxetine treated patients but also suggest that response at the level of miRNA may be drug-specific. 

Many clinical studies of depression, in which human blood was used, focused on single miRNAs or small sets of transcripts. However, other studies have investigated the whole blood miRNome to examine the association between depression, antidepressant treatment, and miRNAs. For instance, Belzeaux and co-workers used small RNA sequencing (sRNA-seq) and found that blood levels of miR-3688 and miR-5695 had predictive validity for treatment-worsening suicidal ideation in depressed patients who received either duloxetine or placebo for 8 weeks [101]. Yrondi and co-workers examined the whole profile of miRNAs in the blood of depressed patients before and after 2 weeks of escitalopram treatment. Using NGS analysis, they found that pharmacological intervention resulted in 33 upregulated and 12 downregulated miRNAs, of which the overexpression of blood miR-185-5p during antidepressant treatment was negatively correlated with nausea, a side effect commonly observed in depressed patients treated with selective serotonin reuptake inhibitors [102]. In another study, Zhao et al. profiled blood miRNA changes between depressed and matched healthy controls. In their study, they used two independent cohorts of patients and found a new and uncharacterized miRNA, pmiR-chr11, that was significantly dysregulated in the blood of depressed patients, and its level was negatively correlated with hippocampal volume [103]. 

### 4.2. Serum/Plasma and Cerebrospinal Fluid (CSF)

Two studies observed an upregulation in serum levels of miR-182 and miR-132 [104] and plasma levels of miR-132 [105] in depressed patients compared to healthy controls. Additionally, both miRNAs regulated BDNF expression in a human neuronal cell model. Additionally, serum miR-182 levels were negatively correlated with serum BDNF levels in depressed patients [104]. In animal studies, chronic unpredictable stress triggered the upregulation of miR-182 expression and decreased BDNF levels in the rat hippocampus [106], while suppression of miR-182 in the same brain structure had antidepressant-like effects [106]. This suggests that miR-182 may be involved in the pathogenesis of depression by decreasing BDNF signaling in humans and in rodents. In another study, Wang et al. observed associations between lower plasma levels of miR-144-5p and depressive symptoms in patients with depression/anxiety, while 8 weeks of personalized antidepressant treatments reversed this trend [107]. miR-144-5p may also play an important role in the hippocampal function of rats experiencing chronic unpredictable stress [108]. In stressed animals, the hippocampal expression of miR-144-5p was significantly downregulated compared to that of the unstressed control group. Lentiviral overexpression of miR-144-5p in the hippocampus of stressed animals had antidepressant properties, mainly by inhibiting the expression of PTP1B protein, which is a known negative regulator of BDNF/TrkB signaling [108]. It has been observed that in depressed patients there is a correlation between serum and cerebrospinal fluid (CSF) levels of several miRNAs, including the downregulation of miR-16-5p [109] and the upregulation of miR-34a-5p, miR-221-3p and let-7d-3p [110,111]. Song et al. have shown significantly lower levels of miR-16-5p in the CSF as well as in serum of depressed patients [109], while another study reported higher levels of miR-16-5p levels in serum of depressed patients who were treated for four weeks with various SSRIs, including escitalopram, fluoxetine, paroxetine and sertraline [100]. In this study however, authors focused on differences between two classes of drugs (i.e., SSRI vs. SNRI) rather than the associations between miRNAs and particular drugs [100]. Interestingly, preclinical studies showed that time-dependent changes in serum (and different brain structures) levels of miR-16-5p may define the resilient phenotype in the mouse chronic mild stress model [112]. In depressed patients, four weeks of antidepressant treatment using different SSRIs normalized serum levels of miR-16-5p [100], while 8 weeks of paroxetine treatment normalized serum levels miR-34a-5p and miR-221-3p [111] (for further details, see Table 2). Interestingly, one of the genes which has been validated to be regulated by miR-16-5p is SLC6A4, the serotonin transporter [99,113], which is the main target of most available antidepressants. Thus, disturbed miR-16-5p-mediated regulation of the serotonin transporter function may be directly related to the development of depression. Gheysarzadeh and colleagues observed downregulated serum levels of miR-1202, miR-16-5p, and miR-135a-5p in depressed patients [114], replicating the results of other studies (for details, see Table 2). Decreased plasma levels of miR-184 [115], let-7g-5p, miR-103a-3p, miR-107, miR-142-3p [116], and miR-134 [117] were also observed in depressed patients. Eight weeks of personalized antidepressant treatment was associated with an increase in plasma levels of miR-134 [117].

### 4.3. Peripheral Blood Mononuclear Cells (PBMCs)

A growing body of evidence indicates that exacerbated activation of inflammatory signaling in the brain leads to behavioral alterations and the development of depression by altering a range of neurocircuitry and neurotransmitter systems [118,119]. The inflammatory mechanism is partially regulated by cytokines released by immunocompetent cells found in the bloodstream, such as PBMCs [120,121]. Moreover, stress—a key factor contributing to the development of depression—can activate proinflammatory responses in PBMCs [122,123]. Since a dialogue between the immune system and the brain is evident, and PBMCs can be easily collected from blood; assessing changes in this group of cells in depressed patients is an attractive method for finding peripheral biomarkers of stress-related mood disorders. It has been reported that depressed patients have altered expression levels of fourteen miRNAs in PBMCs in comparison to healthy subjects [124], of which miR-941 and miR-589 showed stable overexpression in PBMCs, irrespective of antidepressant therapy [124]. Another study reported upregulation of miR-26b, miR-1972, miR-4485, miR-4498, miR-4743 in PBMCs of depressed subjects [125]. This set of miRNAs had good predictive properties in discriminating depressed patients from healthy controls and may potentially be involved in the regulation of the biological pathways related to nervous system functioning [125]. On the other hand, Hung and co-workers observed the downregulated expression of let-7e-5p, miR-21-5p, miR-146a and miR-155 in PBMCs of subjects suffering from depression, while 4 weeks of personalized antidepressant treatment effectively reversed these changes [126]. In animal studies, a lower level of miR-155 in serum has been reported, among other miRNAs, in mice after exposure to restraint stress, however, this study did not examine its expression in PBMCs [127]. Experiencing acute psychological stress has been associated with a significant increase in the expression level of miR-29c-3p in PBMCs of healthy people [128]. Moreover, increased miR-29c-3p expression has been linked with enhanced functional connectivity in the ventromedial prefrontal cortex of participants subjected to psychological stress tasks [128]. Interestingly, it has been shown that depression was linked to increased expression levels of three brain-enriched miRNAs i.e., miR-124-3p [129], miR-34b-5p and miR-34c-5p [130] in PBMCs, and that 8 weeks of personalized antidepressant treatment normalized the expression of miR-124-3p [129]. The significant changes in brain-enriched miRNAs found in PBMCs may suggest that these cells are a good source of potential biological markers that reflect the condition of the stressed brain. However, the mechanisms that may underlie this relationship are mostly unknown and need more research. It should be noted that the overlap between findings of different studies investigating miRNAs in PBMCs of depressed patients is small. Some authors focused on a very small set of, or even singular, miRNAs, while other studies analyzed the whole miRNome. These methodological differences may explain the lack of consistency between studies. 

### 4.4. Extracellular Vesicles

Recently, extracellular vesicles have gained more attention in biomarker research of many different pathologies, including depression [131]. Extracellular vesicles represent a heterogenous group of particles released by cells. Different subpopulations of extracellular vesicles are discriminated based on their size, origin, cargo content or general biochemical composition. Nevertheless, consensus on specific markers of different subpopulations of extracellular vesicles has not yet emerged [132]. Based on size and origin parameters, extracellular vesicles are generally classified to three groups:

1. Large extracellular vesicles (L-EVs), including apoptotic bodies, large oncosomes and microvesicles;
2. Small extracellular vesicles (S-EVs), including exosomes;
3. Extracellular particles (EPs), including exomeres and chromatimers.

L-EVs have a size range of about 100–1000 nm and are of plasma membrane origin [133]. S-EVs are of endocytic origin and their size is within a range of 50-130 nm [133]. EPs on the other hand are small protein-nucleic-acid complexes of unknown cellular compartment and are less than 50 nm in size [134]. All these nanometer-sized vesicles are actively secreted into biofluids, such as plasma, serum, CSF, saliva or urine, by multiple types of cells, and carry specific cargo, which is enriched with small noncoding RNAs, such as miRNAs. Although their exact role is mostly unknown, extracellular vesicles have been implicated in inflammatory diseases [135,136], cancer [137,138], and neurodegeneration [139,140], and their involvement in mental disorders is now under investigation [131]. For instance, high-throughput miRNA sequencing analysis identified a set of 12 upregulated and 20 downregulated miRNAs in serum exosomes of depressed patients [141]. For more details, see Table 2. Subsequent gene enrichment analysis showed that upregulated miRNAs had the potential to regulate the neurotrophin signaling pathway, while the downregulated set of miRNAs was involved in the modulation of pathways related to apoptosis, cell growth, and immune response [141]. In this study, miR-139-5p was found, among others, to be upregulated in serum exosomes of depressed patients, and this finding was also supported by an independent study [142]. Additionally, the level of miR-139-5p in serum exosomes had good performance in discriminating patients with MDD and healthy controls [142]. Interestingly, blood-derived exosomes with increased levels of miR-139-5p collected from depressed subjects had the potential to evoke depressive-like symptoms in mice when administered intravenously [62]. An intranasal injection of a miR-139-5p antagomir alleviated depressive-like behavior in stressed mice [62], which suggests that increased exosomal miR-139-5p levels may mediate, to some degree, the pathogenesis of depression. 

Interestingly, it is possible to extract the extracellular vesicles of brain origin from blood, i.e., those extracellular vesicles that were released from brain cells [93,143]. By studying brain-derived extracellular vesicles (BDEVs), one may obtain molecular information specific to the central nervous system. Indeed, it has been shown that the levels of miR-17-5p in neuron-derived extracellular vesicles found in blood positively correlated with intensification of symptoms of a subthreshold depressive state in humans [143]. Comprehensive molecular analysis of neuron-derived extracellular vesicles found in the plasma of depressed patients treated for 8 weeks with escitalopram demonstrated that these vesicles had brain-specific protein markers and contained miRNAs enriched for brain function [93]. The size of neuron-derived extracellular vesicles found in depressed patients was smaller than in heathy controls and this effect was reversed after antidepressant treatment [93]. Furthermore, time-dependent changes in miR-21-5p, miR-30d-5p and miR-486-5p levels in neuron-derived extracellular vesicles were associated with a different response of depressed patients to antidepressant therapy [93], suggesting that changes in miRNA cargo found in peripherally extracted neuron-derived extracellular vesicles may be a good noninvasive biomarker of antidepressant treatment response. 

### 4.5. Brain-Enriched miRNAs Found in Periphery

One of the most promising findings in this field of research is the detection of miRNAs in the periphery that shows significant correlation with brain imaging changes and the depressive phenotype. For instance, He et al. used a combination of resting-state functional Magnetic Resonance Imaging (fMRI) and peripheral blood miR-9 tests to show that blood levels of neuron-specific miR-9 were higher in depressed patients with a history of childhood maltreatment compared to those observed in healthy controls. Moreover, these alterations were correlated with depressive severity, as well as with changes in amygdala connectivity observed during fMRI scanning in depressed patients. Taken together, their results suggest that miR-9 may play an important role in linking the effects of childhood maltreatment with altered prefrontal-limbic brain connectivity in the depressive phenotype [92]. Furthermore, three other independent studies have reported peripheral changes in another brain-enriched microRNA-miR-124-3p. miR-124-3p regulates neurogenesis and, together with miR-9, is responsible for neuronal differentiation [144,145,146]. miR-124-3p levels were elevated in the plasma [105], serum [85], and peripheral blood mononuclear cells (PBMCs) of depressed patients compared to healthy controls [129]. Moreover, in two reported studies, the miR-124-3p level was normalized (decreased) after 8 weeks of antidepressant treatment [105,129]. Interestingly, other clinical studies have also reported that brain-enriched miRNAs responsible for the regulation of synaptic plasticity, such as miR-128, miR-125a-5p, miR-125b-5p and miR132, were detected in the circulation and their levels correlated with either a depressive state or antidepressant treatment [94,147]. For example, miR-125a-5p was upregulated in the serum of depressed patients [110] and downregulated in plasma after 12 weeks of escitalopram treatment [148]. Additionally, one study reported that 8 weeks of escitalopram treatment decreased plasma levels of miR-132 [105], while another showed an elevated level of the same miR in the whole blood of depressed patients treated with escitalopram for 12 weeks [94]. Both studies used the same drug; however, different durations of treatment and different biological material may explain the inconsistent directions of reported changes. Although the field of research searching for peripheral miRNA biomarkers of depression and antidepressant response is still relatively young, the number of clinical studies focusing on this domain is rising. Together with the growth of this field, we can also observe an increasing consistency in miRNAs reported as associated with depression and/or antidepressant response. Several recent and independent studies have consistently shown that the level of brain-enriched miR-132 was significantly higher in whole blood [149], serum [104] and plasma [105] of people suffering from depression.

## 5. From Changes in the Brain to Changes in the Periphery: A Summary

### 5.1. miR-1202

**Figure 1:**
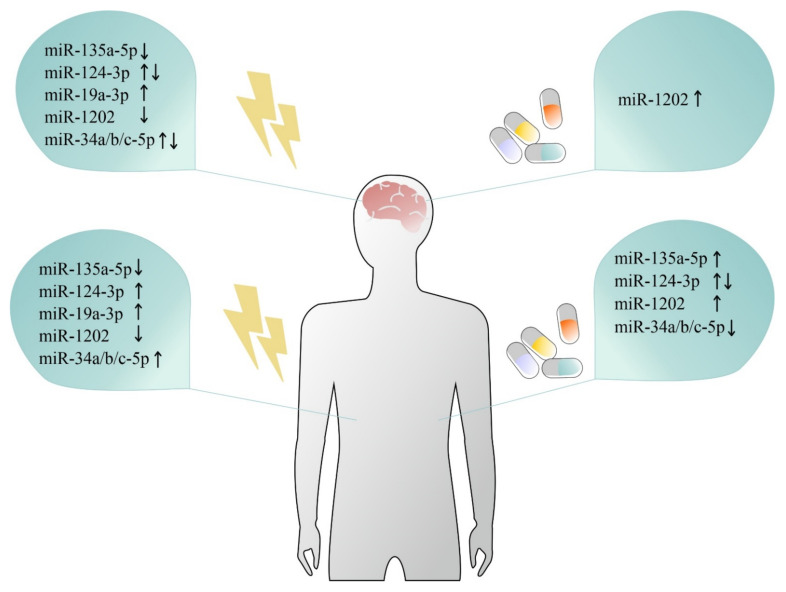
Graphical summary of miRNA changes observed in depression and antidepressant response at the level of brain as well as peripheral tissues. Left panel represents directions of changes in the expression of miRNAs found in brain and periphery in depressed vs. healthy controls. Right panel represents directions of changes in the expression of miRNAs found in brain and periphery in depressed patients after different antidepressant treatments.

miR-1202 is primate-specific and brain-enriched miRNA which regulates GMR4 signaling in the brain. As mentioned earlier, miR-1202 was downregulated in the prefrontal cortex (BA44) of individuals who died during an episode of depression [63]. Antidepressant treatment history also had an effect, as individuals who were not taking antidepressants had lower levels of miR-1202 than those who were treated. Additionally, miR-1202 levels were significantly lower in the blood of depressed patients, and the levels were normalized according to clinical response to 8 weeks of citalopram treatment [63]. These observations were consistent with those found by an independent group [114], which reported that the levels of this miRNA were lower in serum obtained from individuals with depression as compared to healthy controls. Interestingly, the upregulation of blood levels of miR-1202 was also associated with a clinical response to 8 weeks of duloxetine [97] and desvenlafaxine [96]. Further, analysis combining fMRI scans of depressed patients before and after 8 weeks of desvenlafaxine treatment, together with miR-1202 blood levels, revealed that peripheral changes in miR-1202 reflected alterations in brain activity in regions involved in stress perception and interpretation [96]. Together, these observations show consistent miR-1202 dysregulation in post-mortem brain tissue and in the blood of MDD patients (see Figure 1). miR-1202 predictive validity of antidepressant treatment response and its correlations between peripheral variations and functional changes in a depressed living brain suggests involvement in the pathophysiological processes associated with depression. 

### 5.2. miR-124-3p

miR-124-3p—another brain-enriched and neuron-specific miRNA—also appears to play a role in the pathophysiology of depression, and its dysregulation has been observed in the central nervous system and peripheral tissues (see Figure 1). miR-124-3p is involved in the regulation of neurogenesis [162] and synaptic plasticity by targeting memory signaling proteins [163]. An increase in miR-124-3p expression was reported in the frontal cortex (BA46) of individuals who were affected by depression [85], as well as in the plasma [105], PBMCs [129] and serum [85] of antidepressant-free depressed patients. Eight weeks of personalized antidepressant treatment downregulated miR-124-3p levels in PBMCs [129] while its plasma levels were unchanged in depressed patients treated with citalopram [105]. The potential contribution of miR-124-3p to the development of stress-related mood disorders is further suggested by the fact that this miRNA is a regulator of NR3C1 and AMPA selective glutamate receptor 4 (GRIA4), genes significantly involved in various physiological functions in the brain, including synaptic plasticity [85]. Interestingly, the role of miR-124-3p in the regulation of stress-related behaviors was also observed in animal studies. For instance, chronic corticosterone administration induced an increased expression level of miR-124-3p in the prefrontal cortex of rats [85], while inhibition of miR-124-3p by its antagomir in the hippocampus was able to reverse stress-induced depressive-like behavior in mice exposed to chronic corticosterone injections [164] or rats subjected to chronic unpredictable stress [165]. However, the role of miR-124-3p in the regulation of brain function in vivo may be more complex and region specific, since induced genetic miR-124-1 haploinsufficiency also contributed to the development of behavioral deficits, such as impaired prepulse inhibition or social deficits in mice [166], while decreased expression levels of miR-124-3p in the amygdala occurred following exposure of animals to acute stress [167]. In humans, the decreased expression of miR-124-3p in BA44 was significantly associated with depression across multiple studies [65].

### 5.3. miR-19a-3p

Another miRNA that may be involved in the regulation of the biological response to stress at multiple levels is miR-19a-3p. miR-19a-3p has been found to mediate neuronal damage [168,169]. Interestingly, an increase in miR-19a-3p expression was observed in the frontal cortex (BA10) of depressed individuals who died by suicide, while this was not observed in individuals who were depressed but died by causes other than suicide [53]. The similar regulation of miR-19a-3p was found in PBMCs of depressed patients who had serious suicidal ideation [53]. This suggests that miR-19a-3p may be an epigenetic factor that governs molecular mechanisms associated with suicidal phenotypes. 

### 5.4. miR-135a-5p

miR-135a-5p appears to be involved in the systemic regulation of effective coping strategies in response to stress. Decreased expression of miR-135a-5p was observed in the serotonergic raphe nuclei of individuals who died by suicide [84], in the hippocampi of mice and in the prefrontal cortex of rats subjected to chronic mild stress [98,113]. Downregulation of miR-135a-5p in raphe nuclei produced depressive-like behavior while its overexpression resulted in the development of a stress-resilient phenotype in mice [84]. In addition, the pharmacological inhibition of miR-135a-5p in amygdala produced an anxiety-like effect in mice [170]. Total blood levels of miR-135a-5p were significantly reduced in depressed subjects [84,98] and in blood of stressed animals [98]. Cognitive-behavioral therapy (CBT), but not SSRI treatment, reversed these alterations in humans [84]. In animal studies, chronic treatment with miR-135a-5p mimic significantly alleviated depressive-like symptoms in stressed mice [98]. Moreover, baseline miR-135a-5p levels in leukocytes of depressed patients appears to be associated with a better and faster response to antidepressant treatment [151]. Its role in the modulation of the biological stress response may be due to the fact that miR-135a-5p has the potential to orchestrate synaptic plasticity in the brain by targeting complexin-family proteins [170]. The observation that miR-135a-5p expression can be effectively regulated by psychological intervention and may predict the trajectory of antidepressant response suggests its potential role as an endogenous marker for effective stress-coping strategies in humans. 

### 5.5. miR-34 Family

The mammalian miR-34 family includes three mature miRNAs: miR-34a, miR-34b, and miR-34c, which are encoded at two different genomic loci [171]. The miR-34 family targets multiple proteins responsible for cell cycle regulation and thus may promote cell differentiation, including neuronal maturation [171,172,173]. In clinical studies, it has been observed that suicides have a decreased expression of miR-34a-5p in the ACC [77] and an increased expression of miR-34c-5p in BA44 [58] as compared to healthy controls. Interestingly, animal studies showed that lentiviral-mediated overexpression of miR-34c-5p in central amygdala has an anxiolytic effect, which suggested that the upregulation of amygdalar miR-34c-5p may enhance effective coping with stress [59]. On the other hand, chronic fluoxetine administration may increase the expression of miR-34a-5p and decrease the level of the serotonin 5-HT2C receptor in raphe nuclei of treated mice and thus this interaction may elicit the behavioral response to chronic fluoxetine treatment [174]. The modulatory role of miR-34c-5p on the molecular response to stress may be mediated through interaction of this miRNA with its target gene-stress-related corticotropin releasing factor receptor type 1 (CRFR1) in the brain [59]. Mice that carried a complete knockout of genes encoding the miR-34 family expressed a stress-resilient phenotype [175]. Data from animal studies suggest that miR-34c-5p expression is induced by different stressors in many different brain regions; however, its role in regulation of stress response mechanisms is unclear, as both miR-34c-5p overexpression and its complete knockout yielded anxiolytic and pro-resilient effects. A few clinical studies have reported the upregulation of miR-34a-5p in serum [110,126] and CSF [110], as well as miR-34b-5p and miR-34c-5p in PBMCs [130] of depressed patients. Eight weeks of paroxetine and 12 weeks of escitalopram treatment normalized levels of miR-34a-5p in serum [111] and miR-34c-5p levels in whole blood [94]. Interestingly, the levels of blood miR-34c-5p were significantly negatively correlated with language and delayed memory index scores in MDD, but not healthy patients [152], which may suggest that alterations in the expression of the miR-34 family may contribute to decreased cognitive functions observed in depression. 

## 6. Conclusions

Over the past few decades, many biomedical investigations have taken on the challenge of identifying the biological mechanisms underlying depression. Recent advances in the field of biological psychiatry have found that stress-related mood disorders, including depression, are strongly correlated with multiple changes at different molecular levels, such as gene mutations, RNA expression and protein function. However, these observations have not always yielded consistent results in the literature. This phenomenon may result from the fact that miRNAs are considered an additional molecular level, interfering with well-described gene expression pathways. miRNAs, by the post-transcriptional fine-tuning of gene expression, pleiotropism, and high evolutionary conservation, may not only contribute to maintaining homeostatic balance in an organism, but may also play an important biomarker role. Clinical and preclinical studies on the role of miRNAs in the pathophysiology of stress-related mood disorders belong to a relatively new research field that also has different limitations. For instance, there is no gold standard method for measuring miRNA expression. Depression-related miRNA changes have been evaluated in many studies by using various methodologies such as RT-qPCR, microarray or small RNA-seq [176]. Thus, it is crucial to conduct critical interpretations of obtained findings. miRNAs, by their pleiotropic character, may control many important biological pathways in parallel. Therefore, it is important to understand the impact of particular miRNA on the whole set of target genes [177] by using techniques such as RNA-seq screening, rather than focusing on one chosen mRNA target. Currently available bioinformatic tools for the prediction of miRNA-mRNA interaction often use different sets of biological parameters in the prediction process. This leads to a high variability in predicted mRNA targets found by different algorithms. However, combining the results obtained by different prediction algorithms may yield a good library of potential miRNA target genes that can be used for further evaluation. Recent advances in clinical and preclinical studies have pointed out that the miRNA-dependent regulation of gene expression may be an important phenomenon, which is significantly associated with the development of stress-related mood disorders, and may possess the potential to be used as biomarkers for depression and antidepressant treatment response. With that said, further research is needed to clarify the involvement of particular candidate miRNAs in the pathophysiology of depression and antidepressant treatment response. Some miRNA-based therapeutic approaches have been proposed to directly modulate target genes involved in the pathomechanisms of depression. However, our knowledge of the role of miRNAs in depression is still too premature to successfully implement these approaches to the clinic.

## Figures and Tables

**Table 1:** List of post-mortem studies comparing brain miRNA expression data between depressed and psychiatrically healthy subjects.

Ref.	Brain Region	miRNA	Regulation MDD vs. HC	Targeted Gene	Validation of Targeted Gene
[80]	Amygdala	miR-128-3p	Up	DVL1, LEF1, WNT5b	Direct—in vitro
[75]	Lateral habenula	miR-320b-3p, miR-331-3p	Up	N/A	N/A
		miR-323a-3p	Up	ERBB4	Direct—in vitro
	BA24	miR-204-5p, miR-331-3p	Up	N/A	N/A
		miR-323a-3p	Up	ERBB4	Direct—in vitro
[45]	BA9/BA46	miR-484-5p, miR-26b-5p, miR-30d-5p, miR-197-3p	Down	N/A	N/A
[74]	ACC	117 miRNAs (4.16%)	Up	N/A	N/A
		54 miRNAs (2.13%)	Down	N/A	N/A
[44]	BA9	miR-30a-5p, miR-30e-5p	Up	ZDHHC21	Direct—in vitro
		miR-200a-5p	Down	N/A	N/A
[65]	BA44	miR-124-3p	Down *	DDIT	Indirect
[53]	BA10	miR-19a-3p	Up	TNFa	N/A
		miR-20a-5p, miR-92a-1-3p	Down	N/A	N/A
[85]	BA46	miR-124-3p	Up	GRIA3, GRIA4, NR3C1	Direct—in vitro
[78]	Locus coeruleus	miR-17-5p, miR-20b-5p, miR-106a-5p, miR-330-3p, miR-541-3p, miR-582-5p, miR-890, miR-99-3p, miR-550-5p, miR-1179	Up	RELN, GSK-3β, MAOA, CHRM1, PLCB1,GRIK1	Indirect
		miR-409-5p, let-7g-3p, miR-1197	Down		
[64]	BA44	miR-218-5p	Down	DCC	Direct—in vitro
[86]	vPFC	miR-146a-5p, miR-146b-5p, miR-24-3p, miR-425-3p	Up	MAPK/Wnt pathway	Direct—in vitro
[87]	Midbrain	miR-326	Down	UCN1	Direct—in vitro
[77]	ACC	miR-184 and miR-34a-5p	Down	PDE4B, NCOA1, NCOR2	Direct—in vitro
[79]	BLA	miR-511, miR-340	Up	GFRA1	Direct—in vitro
[63]	BA44	miR-1202	Down	GRM4	Direct—in vitro
[84]	Raphe nuclei	miR135a-5p	Down	SLC6A4, HTR1A	Direct—in vitro
[58]	BA44	miR-34c-5p, miR-139-5p, miR-195-5p, miR-320c-3p	Up	STAT1, SMOX	Indirect
[52]	BA10	miR-508-3p, miR-152-3p	Down	N/A	N/A
[43]	BA9	miR-142-5p, miR-137, miR-489, miR-148b, miR-101, miR-324-5p, miR-301a, miR-146a, miR-335, miR-494, miR-20b, miR-376a *, miR-190, miR-155, miR-660, miR-130a, miR-27a, miR-497, miR-10a, miR-20a, miR-142-3p	Down	DNMT3b, VEGFa, BCL-2	Indirect
[51]	BA10	miR-185 *, 491-3p	Up	Trk2- T1	Direct—in vitro

* based on bioinformatic exploratory analysis of currently available microarray datasets.

**Table 2:** List of studies comparing peripheral miRNA expression data in depression and antidepressant treatment.

Ref.	Tissue Source	miRNA	Regulation MDD vs. HC	Antidepressant Treatment	miRNA	Regulation by Treatment	Targeted Gene	Validation of Targeted Gene
[92]	Whole blood	miR-9-5p	Up	-	-	-	-	-
[143]	BDEVs in blood	miR-17-5p	&	-	-	-	-	-
[93]	BDEVs in blood	-	-	Escitalopram (8 weeks)	miR-30d-5p, miR-486-5p	Up #	NR3C1, SIRT1, SERPINE1, RPS6KB1, ATF6, PSEN1	Indirect
[150]	Plasma	miR-19b-3p, miR-3921	Down	-	-	-	-	-
		miR-1180-3p	Up	-	-	-	-	-
[142]	Serum exosomes	miR-139-5p	Up	-	-	-	-	-
[62]	Blood exosomes	miR-139-5p	Up	-	-	-	MAP2	Direct—in vitro
[151]	Buffy coat	-	-	Venlafaxine (12 weeks)	miR-135a-5p	X	-	-
[152]	Whole blood	miR-34b-5p, miR-34c-5p	C *	-	-	-	-	-
[117]	Plasma	miR-134	Down	Personalized (8 weeks)	miR-134	Up	-	-
[102]	Whole blood	-	-	Escitalopram (2 weeks)	33 miRNAs	Up	-	-
					12 miRNAs	Down	-	-
[153]	Serum	miR-221-3p	Up	-	-	-	IRF2	Direct—in vitro
[103]	Whole blood	pmiR-chr11	Up	-	-	-	BRPF1	Direct—in vitro
		miR-1275	Down	-	-	-	-	-
[115]	Plasma	miR-184	Down	-	-	-	-	-
[116]	Plasma	let-7g-5p, miR-103a-3p, miR-107, and miR-142-3p	Down	-	-	-	-	-
[126]	PBMC/Monocytes	let-7e-5p, miR-21-5p, miR-146a, miR-155, miR-146a, miR-155	Down	Personalized (4 weeks)	let-7e-5p, miR-223, miR-146a, miR-155, miR-21-5p, miR-14	Up	TLR4	Indirect
[154]	Plasma	-	-	Duloxetine (6–8 weeks)	miR-23a-3p, miR-16-5p, miR-146a-5p, miR-21-5p	10th percentile	-	-
[101]	Whole blood	-	-	Duloxetine (8 weeks)	miR-3688, miR-5695	Up #	-	-
[114]	Serum	miR-16-5p, miR-135a-5p, miR-1202	Down	-	-	-	-	-
[155]	Whole blood	miR-132-3p	Up	-	-	-	-	-
[156]	Whole blood	miR-132-3p, miR-135b, miR-181b	Up	-	-	-	-	-
		miR-155	Up	-	-	-	SIRT1	Direct—in vitro
[53]	PBMC	miR-19a-3p	Up	-	-	-	TNFa	Indirect
[111]	Serum	miRNA-34a-5p, miRNA-221-3p	Up	Paroxetine (8 weeks)	miRNA-34a-5p, miRNA-221-3p	Down	-	-
		miRNA-451a	Down		miRNA-451a	Up	-	-
[105]	Plasma	miR-132-3p, miR-124-3p	Up	Citalopram (8 weeks)	miR-124-3p	Up	-	-
					miR-132	Down	-	-
[100]	Serum	-	-	SSRI/SNRI (4 weeks)	miR-183, miR-212	Up	-	-
				SSRI (4 weeks)	miR-16-5p	Up	-	-
[97]	Whole blood	miR-1202	Down #	Duloxetine (8 weeks)	miR-1202	Up #	-	-
[85]	Serum	miR-124-3p	Up	-	-	-	-	-
[86]	Whole blood/ Plasma	-	-	Personalized (8 weeks)	miR-146a-5p, miR-146b-5p, miR-24-3p, miR-425-3p, miR-3074-5p	Down #	MAPK/Wnt	Direct—in vitro
[141]	Serum exosomes	miR-1255a, miR-3161, miR-99a-3p, miR-205-5p, miR-26a-1-3p, miR-139-5p, miR-7849-3p, miR-195-5p, miR-125b-2-3p, miR-664a-3p, let-7c-5p, miR-197-3p	Up	-	-	-	-	-
		miR-499a-5p, miR-4732-3p, miR-222-5p, miR-1291, miR-668-3p, miR-425-3p, miR-6511a-3p, iR-145-3p, miR-200a-3p, miR-143-3p, miR-196b-5p, miR-99a-5p, miR-144-3p, miR-584-5p, miR-210-3p, miR-183-5p, miR-107, miR-130b-5p, miR-589-5p, miR-1910-5p	Down	-	-	-	-	-
[96]	Whole blood	-	-	Desvenlafaxine (8 weeks)	miR-1202	Up	-	-
[157]	Whole blood	miR-15a-5p	Up	-	-	-	FKBP5	Direct—in vitro
[130]	PBMC	miR-34b-5p, miR-34c-5p	Up	-	-	-	Notch1	Indirect
[158]	Whole blood	miR-199a-5p, miR-345-5p, miR-330-3p, miR-425-3p, miR-24-3p, miR-29c-5p	Up	-	-	-	-	-
		let-7a-5p, let-7f-5p, let-7d-5p, miR-1915-3p	Down					
[129]	PBMC	miR-124-3p	Up	Personalized (8 weeks)	miR-124-3p	Down	-	-
[159]	Whole blood	let-7b, let-7c	Down	-	-	-	-	-
[128]	PBMC	miR-29c	Up *	-	-	-	-	-
[160]	Whole blood	miR-132-3p	Up	-	-	-	-	-
[148]	Plasma	-	-	Escitalopram (12 weeks)	23 miRNAs	Up	-	-
					17 miRNAs	Down	-	-
[161]	Plasma	miR-451a, miR-17-5p, miR-223-3p	Up	-	-	-	-	-
		miR-320a	Down					
[109]	CSF	miR-16-5p	Down	-	-	-	SLC6A4	Direct—in vitro
	Serum	miR-16-5p	Down	-	-	-		
[110]	CSF	miR-34a-5p, miR-221-3p, let-7d-3p	Up	-	-	-	-	-
		miR-451a	Down					
	Serum	miR-125a-5p, miR-30a-5p, let-7d-3p, miR-34a-5p, miR-221-3p, miR-29b-3p, miR-10a-5p, miR-375	Up					
		miR-155-5p, miR-33a-5p, miR-139-5p, miR-590-5, miR-185-5p, miR-106b-5p, miR-15b-5p, miR-451a	Down					
[149]	Whole blood	miR-132	Up	-	-	-	-	-
[107]	Plasma	miR-144-5p	Down	Personalized (8 weeks)	miR-144-5p	Up	-	-
[95]	Whole blood	miR-335	Down	Citalopram (4 weeks)	miR-335	Up	GRM4	Direct—in vitro
		miR-583, mir-650, miR-708, miR-654	Down					
		miR-644, miR-450b, mir-328, miR-182	Up					
[125]	PBMC	miRNA-26b, miRNA-1972, miRNA-4485, miRNA-4498, miRNA-4743	Up	-	-	-	-	-
[84]	Whole blood	miR-135a-5p	Down	CBT (3 weeks)	miR-135a-5p	Up	SLC6A4, HTR1A	Direct—in vitro
[63]	Whole blood	miR-1202	Down	Citalopram (8 weeks)	miR-1202	Up	GRM4	Direct—in vitro
[104]	Serum	miR-182, miR-132	Up	-	-	-	BDNF	Direct—in vitro
[94]	Whole blood	-	-	Escitalopram (12 weeks)	28 miRNAs	Up	-	
					miR-34c-5p, miR-770-5p	Down		
[124]	PBMC	miR-589, miR-579, miR-941, miR-133a, miR-494, miR-107, miR-148a, miR-652, miR-425-3p	Up	Personalized (8 weeks)	miR-20b-3p, miR-433, miR-409-3p, miR-410, miR-485-3p, miR-133a, miR-145	Up	-	-
		miR-517b, miR-636, miR-1243, miR-381, miR-200c	Down		miR-331-5p	Down		

&: positive correlation with PHQ-9 scores in subthreshold depression. X: higher level correlated with better response to antidepressant therapy. C *: positive correlation with worsening of cognitive functions in MDD but not in Control subjects. #: in responders. Up *: in healthy subjects experiencing sustained stress after psychological task. CBT: cognitive-behavioral therapy.

## Data Availability

This study did not report any data.

## References

[1] Manji H.K., Drevets W.C., Charney D.S. (2001). The Cellular Neurobiology of Depression. Nat. Med..

[2] Jeon S., Kim Y.-K. (2016). Molecular Neurobiology and Promising New Treatment in Depression. Int. J. Mol. Sci..

[3] Albert P.R., Benkelfat C., Descarries L. (2012). The Neurobiology of Depression—Revisiting the Serotonin Hypothesis. I. Cellular and Molecular Mechanisms. Philos. Trans. R. Soc. B Biol. Sci..

[4] Chandley M.J., Ordway G.A., Dwivedi Y. (2012). Noradrenergic Dysfunction in Depression and Suicide. The Neurobiological Basis of Suicide.

[5] Belujon P., Grace A.A. (2017). Dopamine System Dysregulation in Major Depressive Disorders. Int. J. Neuropsychopharmacol..

[6] Liu Y., Zhao J., Guo W. (2018). Emotional Roles of Mono-Aminergic Neurotransmitters in Major Depressive Disorder and Anxiety Disorders. Front. Psychol..

[7] Martin-Hernández D., Pereira M.P., Tendilla-Beltrán H., Madrigal J.L.M., García-Bueno B., Leza J.C., Caso J.R. (2019). Modulation of Monoaminergic Systems by Antidepressants in the Frontal Cortex of Rats After Chronic Mild Stress Exposure. Mol. Neurobiol..

[8] Nestler E.J., Carlezon W.A. (2006). The Mesolimbic Dopamine Reward Circuit in Depression. Biol. Psychiatry.

[9] Michely J., Eldar E., Martin I.M., Dolan R.J. (2020). A Mechanistic Account of Serotonin’s Impact on Mood. Nat. Commun..

[10] Rush A.J., Trivedi M.H., Wisniewski S.R., Stewart J.W., Nierenberg A.A., Thase M.E., Ritz L., Biggs M.M., Warden D., Luther J.F. (2006). Bupropion-SR, Sertraline, or Venlafaxine-XR after Failure of SSRIs for Depression. N. Engl. J. Med..

[11] De Carlo V., Calati R., Serretti A. (2016). Socio-Demographic and Clinical Predictors of Non-Response/Non-Remission in Treatment Resistant Depressed Patients: A Systematic Review. Psychiatry Res..

[12] Uher R. (2008). The Implications of Gene–Environment Interactions in Depression: Will Cause Inform Cure?. Mol. Psychiatry.

[13] Lin E., Tsai S.-J. (2019). Epigenetics and Depression: An Update. Psychiatry Investig..

[14] Kwong A.S.F., López-López J.A., Hammerton G., Manley D., Timpson N.J., Leckie G., Pearson R.M. (2019). Genetic and Environmental Risk Factors Associated With Trajectories of Depression Symptoms From Adolescence to Young Adulthood. JAMA Netw. Open.

[15] McIntosh A.M., Sullivan P.F., Lewis C.M. (2019). Uncovering the Genetic Architecture of Major Depression. Neuron.

[16] McEwen B.S., Bowles N.P., Gray J.D., Hill M.N., Hunter R.G., Karatsoreos I.N., Nasca C. (2015). Mechanisms of Stress in the Brain. Nat. Neurosci..

[17] Howard D.M., Adams M.J., Clarke T.-K., Hafferty J.D., Gibson J., Shirali M., Coleman J.R.I., Hagenaars S.P., Ward J., Wigmore E.M. (2019). Genome-Wide Meta-Analysis of Depression Identifies 102 Independent Variants and Highlights the Importance of the Prefrontal Brain Regions. Nat. Neurosci..

[18] Khan A.R., Geiger L., Wiborg O., Czéh B. (2020). Stress-Induced Morphological, Cellular and Molecular Changes in the Brain—Lessons Learned from the Chronic Mild Stress Model of Depression. Cells.

[19] Dziedzicka-Wasylewska M., Solich J., Korlatowicz A., Faron-Górecka A. (2021). What Do the Animal Studies of Stress Resilience Teach Us?. Cells.

[20] Lee R.C., Feinbaum R.L., Ambros V. (1993). The *C. elegans* Heterochronic Gene Lin-4 Encodes Small RNAs with Antisense Complementarity to Lin-14. Cell.

[21] Fire A., Xu S., Montgomery M.K., Kostas S.A., Driver S.E., Mello C.C. (1998). Potent and Specific Genetic Interference by Double-Stranded RNA in *Caenorhabditis elegans*. Nature.

[22] Kozomara A., Griffiths-Jones S. (2014). MiRBase: Annotating High Confidence MicroRNAs Using Deep Sequencing Data. Nucleic Acids Res..

[23] Kozomara A., Birgaoanu M., Griffiths-Jones S. (2019). MiRBase: From MicroRNA Sequences to Function. Nucleic Acids Res..

[24] Artigas F., Celada P., Bortolozzi A. (2018). Can We Increase the Speed and Efficacy of Antidepressant Treatments? Part II. Glutamatergic and RNA Interference Strategies. Eur. Neuropsychopharmacol..

[25] Xue Q., Yu C., Wang Y., Liu L., Zhang K., Fang C., Liu F., Bian G., Song B., Yang A. (2016). MiR-9 and MiR-124 Synergistically Affect Regulation of Dendritic Branching via the AKT/GSK3β Pathway by Targeting Rap2a. Sci. Rep..

[26] Friedman R.C., Farh K.K.-H., Burge C.B., Bartel D.P. (2008). Most Mammalian MRNAs Are Conserved Targets of MicroRNAs. Genome Res..

[27] Kim Y.-K., Kim B., Kim V.N. (2016). Re-Evaluation of the Roles of *DROSHA*, *Exportin 5*, and *DICER* in MicroRNA Biogenesis. Proc. Natl. Acad. Sci. USA.

[28] Martinez N.J., Gregory R.I. (2013). Argonaute2 Expression Is Post-Transcriptionally Coupled to MicroRNA Abundance. RNA.

[29] O’Brien J., Hayder H., Zayed Y., Peng C. (2018). Overview of MicroRNA Biogenesis, Mechanisms of Actions, and Circulation. Front. Endocrinol..

[30] Riffo-Campos Á., Riquelme I., Brebi-Mieville P. (2016). Tools for Sequence-Based MiRNA Target Prediction: What to Choose?. Int. J. Mol. Sci..

[31] O’Connor R.M., Dinan T.G., Cryan J.F. (2012). Little Things on Which Happiness Depends: MicroRNAs as Novel Therapeutic Targets for the Treatment of Anxiety and Depression. Mol. Psychiatry.

[32] Tian X., Zhang H., Zhang J., Xing J. (2016). Reciprocal Regulation between MRNA and MicroRNA Enables a Bistable Switch That Directs Cell Fate Decisions. FEBS Lett..

[33] Rajman M., Schratt G. (2017). MicroRNAs in Neural Development: From Master Regulators to Fine-Tuners. Development.

[34] Venø M.T., Reschke C.R., Morris G., Connolly N.M.C., Su J., Yan Y., Engel T., Jimenez-Mateos E.M., Harder L.M., Pultz D. (2020). A Systems Approach Delivers a Functional MicroRNA Catalog and Expanded Targets for Seizure Suppression in Temporal Lobe Epilepsy. Proc. Natl. Acad. Sci. USA.

[35] Park C.S., Tang S.-J. (2009). Regulation of MicroRNA Expression by Induction of Bidirectional Synaptic Plasticity. J. Mol. Neurosci..

[36] Gurwitz D. (2019). Genomics and the Future of Psychopharmacology: MicroRNAs Offer Novel Therapeutics. Dialogues Clin. Neurosci..

[37] Babiloni C., Ferretti A., Del Gratta C., Carducci F., Vecchio F., Romani G.L., Rossini P.M. (2005). Human Cortical Responses during One-Bit Delayed-Response Tasks: An FMRI Study. Brain Res. Bull..

[38] Pirau L., Lui F. (2021). Frontal Lobe Syndrome. StatPearls.

[39] Fischer M., Moscovitch M., Alain C. (2021). A Systematic Review and Meta-analysis of Memory-guided Attention: Frontal and Parietal Activation Suggests Involvement of Fronto-parietal Networks. WIREs Cogn. Sci..

[40] Lane R.D., Reiman E.M., Bradley M.M., Lang P.J., Ahern G.L., Davidson R.J., Schwartz G.E. (1997). Neuroanatomical Correlates of Pleasant and Unpleasant Emotion. Neuropsychologia.

[41] Ho A.M.-C., Winham S.J., Armasu S.M., Blacker C.J., Millischer V., Lavebratt C., Overholser J.C., Jurjus G.J., Dieter L., Mahajan G. (2019). Genome-Wide DNA Methylomic Differences between Dorsolateral Prefrontal and Temporal Pole Cortices of Bipolar Disorder. J. Psychiatr. Res..

[42] Herwig U., Padberg F., Unger J., Spitzer M., Schönfeldt-Lecuona C. (2001). Transcranial Magnetic Stimulation in Therapy Studies: Examination of the Reliability of “Standard” Coil Positioning by Neuronavigation. Biol. Psychiatry.

[43] Smalheiser N.R., Lugli G., Rizavi H.S., Torvik V.I., Turecki G., Dwivedi Y. (2012). MicroRNA Expression Is Down-Regulated and Reorganized in Prefrontal Cortex of Depressed Suicide Subjects. PLoS ONE.

[44] Gorinski N., Bijata M., Prasad S., Wirth A., Abdel Galil D., Zeug A., Bazovkina D., Kondaurova E., Kulikova E., Ilchibaeva T. (2019). Attenuated Palmitoylation of Serotonin Receptor 5-HT1A Affects Receptor Function and Contributes to Depression-like Behaviors. Nat. Commun..

[45] Wingo T.S., Yang J., Fan W., Min Canon S., Gerasimov E.S., Lori A., Logsdon B., Yao B., Seyfried N.T., Lah J.J. (2020). Brain MicroRNAs Associated with Late-Life Depressive Symptoms Are Also Associated with Cognitive Trajectory and Dementia. Npj Genomic Med..

[46] Babiloni C., Vecchio F., Bares M., Brazdil M., Nestrasil I., Eusebi F., Maria Rossini P., Rektor I. (2008). Functional Coupling between Anterior Prefrontal Cortex (BA10) and Hand Muscle Contraction during Intentional and Imitative Motor Acts. NeuroImage.

[47] Semendeferi K., Armstrong E., Schleicher A., Zilles K., Van Hoesen G.W. (2001). Prefrontal Cortex in Humans and Apes: A Comparative Study of Area 10. Am. J. Phys. Anthropol..

[48] Katayama N., Nakagawa A., Umeda S., Terasawa Y., Kurata C., Tabuchi H., Kikuchi T., Mimura M. (2019). Frontopolar Cortex Activation Associated with Pessimistic Future-Thinking in Adults with Major Depressive Disorder. NeuroImage Clin..

[49] Rogers R.D., Owen A.M., Middleton H.C., Williams E.J., Pickard J.D., Sahakian B.J., Robbins T.W. (1999). Choosing between Small, Likely Rewards and Large, Unlikely Rewards Activates Inferior and Orbital Prefrontal Cortex. J. Neurosci..

[50] Pizzagalli D.A., Iosifescu D., Hallett L.A., Ratner K.G., Fava M. (2008). Reduced Hedonic Capacity in Major Depressive Disorder: Evidence from a Probabilistic Reward Task. J. Psychiatr. Res..

[51] Maussion G., Yang J., Yerko V., Barker P., Mechawar N., Ernst C., Turecki G. (2012). Regulation of a Truncated Form of Tropomyosin-Related Kinase B (TrkB) by Hsa-MiR-185* in Frontal Cortex of Suicide Completers. PLoS ONE.

[52] Smalheiser N.R., Lugli G., Zhang H., Rizavi H., Cook E.H., Dwivedi Y. (2014). Expression of MicroRNAs and Other Small RNAs in Prefrontal Cortex in Schizophrenia, Bipolar Disorder and Depressed Subjects. PLoS ONE.

[53] Wang Q., Roy B., Turecki G., Shelton R.C., Dwivedi Y. (2018). Role of Complex Epigenetic Switching in Tumor Necrosis Factor-α Upregulation in the Prefrontal Cortex of Suicide Subjects. Am. J. Psychiatry.

[54] Kim T., Valera E., Desplats P. (2019). Alterations in Striatal MicroRNA-MRNA Networks Contribute to Neuroinflammation in Multiple System Atrophy. Mol. Neurobiol..

[55] Stewart C., Riedel K. (2016). Managing Speech and Language Deficits after Stroke. Stroke Rehabilitation.

[56] Graïc J.-M., Peruffo A., Corain L., Centelleghe C., Granato A., Zanellato E., Cozzi B. (2020). Asymmetry in the Cytoarchitecture of the Area 44 Homolog of the Brain of the Chimpanzee Pan Troglodytes. Front. Neuroanat..

[57] Wildgruber D., Riecker A., Hertrich I., Erb M., Grodd W., Ethofer T., Ackermann H. (2005). Identification of Emotional Intonation Evaluated by FMRI. NeuroImage.

[58] Lopez J.P., Fiori L.M., Gross J.A., Labonte B., Yerko V., Mechawar N., Turecki G. (2014). Regulatory Role of MiRNAs in Polyamine Gene Expression in the Prefrontal Cortex of Depressed Suicide Completers. Int. J. Neuropsychopharmacol..

[59] Haramati S., Navon I., Issler O., Ezra-Nevo G., Gil S., Zwang R., Hornstein E., Chen A. (2011). MicroRNA as Repressors of Stress-Induced Anxiety: The Case of Amygdalar MiR-34. J. Neurosci..

[60] Song W., Shen Y., Zhang Y., Peng S., Zhang R., Ning A., Li H., Li X., Lin G.N., Yu S. (2019). Expression Alteration of MicroRNAs in Nucleus Accumbens Is Associated with Chronic Stress and Antidepressant Treatment in Rats. BMC Med. Inform. Decis. Mak..

[61] McKibben L.A., Dwivedi Y. (2021). Early-Life Stress Induces Genome-Wide Sex-Dependent MiRNA Expression and Correlation across Limbic Brain Areas in Rats. Epigenomics.

[62] Wei Z.-X., Xie G.-J., Mao X., Zou X.-P., Liao Y.-J., Liu Q.-S., Wang H., Cheng Y. (2020). Exosomes from Patients with Major Depression Cause Depressive-like Behaviors in Mice with Involvement of MiR-139-5p-Regulated Neurogenesis. Neuropsychopharmacology.

[63] Lopez J.P., Lim R., Cruceanu C., Crapper L., Fasano C., Labonte B., Maussion G., Yang J.P., Yerko V., Vigneault E. (2014). MiR-1202 Is a Primate-Specific and Brain-Enriched MicroRNA Involved in Major Depression and Antidepressant Treatment. Nat. Med..

[64] Torres-Berrío A., Lopez J.P., Bagot R.C., Nouel D., Dal Bo G., Cuesta S., Zhu L., Manitt C., Eng C., Cooper H.M. (2017). DCC Confers Susceptibility to Depression-like Behaviors in Humans and Mice and Is Regulated by MiR-218. Biol. Psychiatry.

[65] Wang Q., Zhao G., Yang Z., Liu X., Xie P. (2017). Downregulation of MicroRNA-124-3p Suppresses the MTOR Signaling Pathway by Targeting DDIT4 in Males with Major Depressive Disorder. Int. J. Mol. Med..

[66] Stevens F.L., Hurley R.A., Taber K.H. (2011). Anterior Cingulate Cortex: Unique Role in Cognition and Emotion. J. Neuropsychiatry Clin. Neurosci..

[67] Gasquoine P.G. (2013). Localization of Function in Anterior Cingulate Cortex: From Psychosurgery to Functional Neuroimaging. Neurosci. Biobehav. Rev..

[68] Palomero-Gallagher N., Mohlberg H., Zilles K., Vogt B. (2008). Cytology and Receptor Architecture of Human Anterior Cingulate Cortex. J. Comp. Neurol..

[69] Davis K.D. (2005). Human Anterior Cingulate Cortex Neurons Encode Cognitive and Emotional Demands. J. Neurosci..

[70] Casey B.J., Thomas K.M., Welsh T.F., Badgaiyan R.D., Eccard C.H., Jennings J.R., Crone E.A. (2000). Dissociation of Response Conflict, Attentional Selection, and Expectancy with Functional Magnetic Resonance Imaging. Proc. Natl. Acad. Sci. USA.

[71] Tripp A., Oh H., Guilloux J.-P., Martinowich K., Lewis D.A., Sibille E. (2012). Brain-Derived Neurotrophic Factor Signaling and Subgenual Anterior Cingulate Cortex Dysfunction in Major Depressive Disorder. Am. J. Psychiatry.

[72] Ho T.C., Sacchet M.D., Connolly C.G., Margulies D.S., Tymofiyeva O., Paulus M.P., Simmons A.N., Gotlib I.H., Yang T.T. (2017). Inflexible Functional Connectivity of the Dorsal Anterior Cingulate Cortex in Adolescent Major Depressive Disorder. Neuropsychopharmacology.

[73] Kozel F.A., Rao U., Lu H., Nakonezny P.A., Grannemann B., McGregor T., Croarkin P.E., Mapes K.S., Tamminga C.A., Trivedi M.H. (2011). Functional Connectivity of Brain Structures Correlates with Treatment Outcome in Major Depressive Disorder. Front. Psychiatry.

[74] Yoshino Y., Roy B., Dwivedi Y. (2020). Altered MiRNA Landscape of the Anterior Cingulate Cortex Is Associated with Potential Loss of Key Neuronal Functions in Depressed Brain. Eur. Neuropsychopharmacol..

[75] Fiori L.M., Kos A., Lin R., Théroux J.-F., Lopez J.P., Kühne C., Eggert C., Holzapfel M., Huettl R.-E., Mechawar N. (2020). MiR-323a Regulates ERBB4 and Is Involved in Depression. Mol. Psychiatry.

[76] Zucchi F.C.R., Yao Y., Ward I.D., Ilnytskyy Y., Olson D.M., Benzies K., Kovalchuk I., Kovalchuk O., Metz G.A.S. (2013). Maternal Stress Induces Epigenetic Signatures of Psychiatric and Neurological Diseases in the Offspring. PLoS ONE.

[77] Azevedo J.A., Carter B.S., Meng F., Turner D.L., Dai M., Schatzberg A.F., Barchas J.D., Jones E.G., Bunney W.E., Myers R.M. (2016). The MicroRNA Network Is Altered in Anterior Cingulate Cortex of Patients with Unipolar and Bipolar Depression. J. Psychiatr. Res..

[78] Roy B., Wang Q., Palkovits M., Faludi G., Dwivedi Y. (2017). Altered MiRNA Expression Network in Locus Coeruleus of Depressed Suicide Subjects. Sci. Rep..

[79] Maheu M., Lopez J.P., Crapper L., Davoli M.A., Turecki G., Mechawar N. (2015). MicroRNA Regulation of Central Glial Cell Line-Derived Neurotrophic Factor (GDNF) Signalling in Depression. Transl. Psychiatry.

[80] Roy B., Dunbar M., Agrawal J., Allen L., Dwivedi Y. (2020). Amygdala-Based Altered MiRNome and Epigenetic Contribution of MiR-128-3p in Conferring Susceptibility to Depression-Like Behavior via Wnt Signaling. Int. J. Neuropsychopharmacol..

[81] Zheng D., Sabbagh J.J., Blair L.J., Darling A.L., Wen X., Dickey C.A. (2016). MicroRNA-511 Binds to FKBP5 MRNA, Which Encodes a Chaperone Protein, and Regulates Neuronal Differentiation. J. Biol. Chem..

[82] Xu J., Wang R., Liu Y., Wang W., Liu D., Jiang H., Pan F. (2019). Short- and Long-Term Alterations of FKBP5-GR and Specific MicroRNAs in the Prefrontal Cortex and Hippocampus of Male Rats Induced by Adolescent Stress Contribute to Depression Susceptibility. Psychoneuroendocrinology.

[83] Maurel O.M., Torrisi S.A., Barbagallo C., Purrello M., Salomone S., Drago F., Ragusa M., Leggio G.M. (2021). Dysregulation of MiR-15a-5p, MiR-497a-5p and MiR-511-5p Is Associated with Modulation of BDNF and FKBP5 in Brain Areas of PTSD-Related Susceptible and Resilient Mice. Int. J. Mol. Sci..

[84] Issler O., Haramati S., Paul E.D., Maeno H., Navon I., Zwang R., Gil S., Mayberg H.S., Dunlop B.W., Menke A. (2014). MicroRNA 135 Is Essential for Chronic Stress Resiliency, Antidepressant Efficacy, and Intact Serotonergic Activity. Neuron.

[85] Roy B., Dunbar M., Shelton R.C., Dwivedi Y. (2017). Identification of MicroRNA-124-3p as a Putative Epigenetic Signature of Major Depressive Disorder. Neuropsychopharmacology.

[86] Lopez J.P., Fiori L.M., Cruceanu C., Lin R., Labonte B., Cates H.M., Heller E.A., Vialou V., Ku S.M., Gerald C. (2017). MicroRNAs 146a/b-5 and 425-3p and 24-3p Are Markers of Antidepressant Response and Regulate MAPK/Wnt-System Genes. Nat. Commun..

[87] Aschrafi A., Verheijen J.M., Gordebeke P.M., Loohuis N.F.O., Menting K., Jager A., Palkovits M., Geenen B., Kos A., Martens G.J.M. (2016). MicroRNA-326 Acts as a Molecular Switch in the Regulation of Midbrain Urocortin 1 Expression. J. Psychiatry Neurosci..

[88] Yuan H., Mischoulon D., Fava M., Otto M.W. (2018). Circulating MicroRNAs as Biomarkers for Depression: Many Candidates, Few Finalists. J. Affect. Disord..

[89] Glinge C., Clauss S., Boddum K., Jabbari R., Jabbari J., Risgaard B., Tomsits P., Hildebrand B., Kääb S., Wakili R. (2017). Stability of Circulating Blood-Based MicroRNAs—Pre-Analytic Methodological Considerations. PLoS ONE.

[90] Mompeón A., Ortega-Paz L., Vidal-Gómez X., Costa T.J., Pérez-Cremades D., Garcia-Blas S., Brugaletta S., Sanchis J., Sabate M., Novella S. (2020). Disparate MiRNA Expression in Serum and Plasma of Patients with Acute Myocardial Infarction: A Systematic and Paired Comparative Analysis. Sci. Rep..

[91] Das Gupta S., Ciszek R., Heiskanen M., Lapinlampi N., Kukkonen J., Leinonen V., Puhakka N., Pitkänen A. (2021). Plasma MiR-9-3p and MiR-136-3p as Potential Novel Diagnostic Biomarkers for Experimental and Human Mild Traumatic Brain Injury. Int. J. Mol. Sci..

[92] He C., Bai Y., Wang Z., Fan D., Wang Q., Liu X., Zhang H., Zhang H., Zhang Z., Yao H. (2021). Identification of MicroRNA-9 Linking the Effects of Childhood Maltreatment on Depression Using Amygdala Connectivity. NeuroImage.

[93] Saeedi S., Nagy C., Ibrahim P., Théroux J.-F., Wakid M., Fiori L.M., Yang J., Rotzinger S., Foster J.A., Mechawar N. (2021). Neuron-Derived Extracellular Vesicles Enriched from Plasma Show Altered Size and MiRNA Cargo as a Function of Antidepressant Drug Response. Mol. Psychiatry.

[94] Bocchio-Chiavetto L., Maffioletti E., Bettinsoli P., Giovannini C., Bignotti S., Tardito D., Corrada D., Milanesi L., Gennarelli M. (2013). Blood MicroRNA Changes in Depressed Patients during Antidepressant Treatment. Eur. Neuropsychopharmacol..

[95] Li J., Meng H., Cao W., Qiu T. (2015). MiR-335 Is Involved in Major Depression Disorder and Antidepressant Treatment through Targeting GRM4. Neurosci. Lett..

[96] Lopez J.P., Pereira F., Richard-Devantoy S., Berlim M., Chachamovich E., Fiori L.M., Niola P., Turecki G., Jollant F. (2017). Co-Variation of Peripheral Levels of MiR-1202 and Brain Activity and Connectivity During Antidepressant Treatment. Neuropsychopharmacology.

[97] Fiori L.M., Lopez J.P., Richard-Devantoy S., Berlim M., Chachamovich E., Jollant F., Foster J., Rotzinger S., Kennedy S.H., Turecki G. (2017). Investigation of MiR-1202, MiR-135a, and MiR-16 in Major Depressive Disorder and Antidepressant Response. Int. J. Neuropsychopharmacol..

[98] Ding Y., Zhong M., Qiu B., Liu C., Wang J., Liang J. (2021). Abnormal Expression of MiR-135a in Patients with Depression and Its Possible Involvement in the Pathogenesis of the Condition. Exp. Ther. Med..

[99] Baudry A., Mouillet-Richard S., Schneider B., Launay J.-M., Kellermann O. (2010). MiR-16 Targets the Serotonin Transporter: A New Facet for Adaptive Responses to Antidepressants. Science.

[100] Lin C.-C., Tsai M.-C., Lee C.-T., Sun M.-H., Huang T.-L. (2018). Antidepressant Treatment Increased Serum MiR- 183 and MiR-212 Levels in Patients with Major Depressive Disorder. Psychiatry Res..

[101] Belzeaux R., Fiori L.M., Lopez J.P., Boucekine M., Boyer L., Blier P., Farzan F., Frey B.N., Giacobbe P., Lam R.W. (2019). Predicting Worsening Suicidal Ideation With Clinical Features and Peripheral Expression of Messenger RNA and MicroRNA During Antidepressant Treatment. J. Clin. Psychiatry.

[102] Yrondi A., Fiori L.M., Frey B.N., Lam R.W., MacQueen G.M., Milev R., Müller D.J., Foster J.A., Kennedy S.H., Turecki G. (2020). Association Between Side Effects and Blood MicroRNA Expression Levels and Their Targeted Pathways in Patients With Major Depressive Disorder Treated by a Selective Serotonin Reuptake Inhibitor, Escitalopram: A CAN-BIND-1 Report. Int. J. Neuropsychopharmacol..

[103] Zhao L., Yang X., Cui L., Wei J., Ni P., Li M., Wang Y., He Y., Li X., Liang S. (2019). Increased Expression of a Novel MiRNA in Peripheral Blood Is Negatively Correlated with Hippocampal Volume in Patients with Major Depressive Disorder. J. Affect. Disord..

[104] Li Y.-J., Xu M., Gao Z.-H., Wang Y.-Q., Yue Z., Zhang Y.-X., Li X.-X., Zhang C., Xie S.-Y., Wang P.-Y. (2013). Alterations of Serum Levels of BDNF-Related MiRNAs in Patients with Depression. PLoS ONE.

[105] Fang Y., Qiu Q., Zhang S., Sun L., Li G., Xiao S., Li X. (2018). Changes in MiRNA-132 and MiR-124 Levels in Non-Treated and Citalopram-Treated Patients with Depression. J. Affect. Disord..

[106] Li Y., Li S., Yan J., Wang D., Yin R., Zhao L., Zhu Y., Zhu X. (2016). MiR-182 (MicroRNA-182) Suppression in the Hippocampus Evokes Antidepressant-like Effects in Rats. Prog. Neuropsychopharmacol. Biol. Psychiatry.

[107] Wang X., Sundquist K., Hedelius A., Palmér K., Memon A.A., Sundquist J. (2015). Circulating MicroRNA-144-5p Is Associated with Depressive Disorders. Clin. Epigenetics.

[108] Li Y., Wang N., Pan J., Wang X., Zhao Y., Guo Z. (2021). Hippocampal MiRNA-144 Modulates Depressive-Like Behaviors in Rats by Targeting PTP1B. Neuropsychiatr. Dis. Treat..

[109] Song M.-F., Dong J.-Z., Wang Y.-W., He J., Ju X., Zhang L., Zhang Y.-H., Shi J.-F., Lv Y.-Y. (2015). CSF MiR-16 Is Decreased in Major Depression Patients and Its Neutralization in Rats Induces Depression-like Behaviors via a Serotonin Transmitter System. J. Affect. Disord..

[110] Wan Y., Liu Y., Wang X., Wu J., Liu K., Zhou J., Liu L., Zhang C. (2015). Identification of Differential MicroRNAs in Cerebrospinal Fluid and Serum of Patients with Major Depressive Disorder. PLoS ONE.

[111] Kuang W.-H., Dong Z.-Q., Tian L.-T., Li J. (2018). MicroRNA-451a, MicroRNA-34a-5p, and MicroRNA-221-3p as Predictors of Response to Antidepressant Treatment. Braz. J. Med. Biol. Res..

[112] Zurawek D., Kusmider M., Faron-Gorecka A., Gruca P., Pabian P., Kolasa M., Solich J., Szafran-Pilch K., Papp M., Dziedzicka-Wasylewska M. (2016). Time-Dependent MiR-16 Serum Fluctuations Together with Reciprocal Changes in the Expression Level of MiR-16 in Mesocortical Circuit Contribute to Stress Resilient Phenotype in Chronic Mild Stress—An Animal Model of Depression. Eur. Neuropsychopharmacol..

[113] Zurawek D., Kusmider M., Faron-Gorecka A., Gruca P., Pabian P., Solich J., Kolasa M., Papp M., Dziedzicka-Wasylewska M. (2017). Reciprocal MicroRNA Expression in Mesocortical Circuit and Its Interplay with Serotonin Transporter Define Resilient Rats in the Chronic Mild Stress. Mol. Neurobiol..

[114] Gheysarzadeh A., Sadeghifard N., Afraidooni L., Pooyan F., Mofid M., Valadbeigi H., Bakhtiari H., Keikhavani S. (2018). Serum-Based MicroRNA Biomarkers for Major Depression: MiR-16, MiR-135a, and MiR-1202. J. Res. Med. Sci..

[115] Mendes-Silva A.P., Fujimura P.T., Silva J.R.d.C., Teixeira A.L., Vieira E.M., Guedes P.H.G., Barroso L.S.S., Nicolau M.d.S., Ferreira J.D.R., Bertola L. (2019). Brain-Enriched MicroRNA-184 Is Downregulated in Older Adults with Major Depressive Disorder: A Translational Study. J. Psychiatr. Res..

[116] Van der Auwera S., Ameling S., Wittfeld K., d’Harcourt Rowold E., Nauck M., Völzke H., Suhre K., Najafi-Shoushtari H., Methew J., Ramachandran V. (2019). Association of Childhood Traumatization and Neuropsychiatric Outcomes with Altered Plasma Micro RNA-Levels. Neuropsychopharmacology.

[117] Zhang H., Liu X., Chen J., Cheng K., Bai S.-J., Zheng P., Zhou C., Wang W., Wang H., Zhong L. (2020). Circulating MicroRNA 134 Sheds Light on the Diagnosis of Major Depressive Disorder. Transl. Psychiatry.

[118] Felger J.C., Lotrich F.E. (2013). Inflammatory Cytokines in Depression: Neurobiological Mechanisms and Therapeutic Implications. Neuroscience.

[119] Miller A.H., Maletic V., Raison C.L. (2009). Inflammation and Its Discontents: The Role of Cytokines in the Pathophysiology of Major Depression. Biol. Psychiatry.

[120] Dowlati Y., Herrmann N., Swardfager W., Liu H., Sham L., Reim E.K., Lanctôt K.L. (2010). A Meta-Analysis of Cytokines in Major Depression. Biol. Psychiatry.

[121] Cyranowski J.M., Marsland A.L., Bromberger J.T., Whiteside T.L., Chang Y., Matthews K.A. (2007). Depressive Symptoms and Production of Proinflammatory Cytokines by Peripheral Blood Mononuclear Cells Stimulated in Vitro. Brain. Behav. Immun..

[122] Bierhaus A., Wolf J., Andrassy M., Rohleder N., Humpert P.M., Petrov D., Ferstl R., von Eynatten M., Wendt T., Rudofsky G. (2003). A Mechanism Converting Psychosocial Stress into Mononuclear Cell Activation. Proc. Natl. Acad. Sci. USA.

[123] Pace T.W.W., Mletzko T.C., Alagbe O., Musselman D.L., Nemeroff C.B., Miller A.H., Heim C.M. (2006). Increased Stress-Induced Inflammatory Responses in Male Patients With Major Depression and Increased Early Life Stress. Am. J. Psychiatry.

[124] Belzeaux R., Bergon A., Jeanjean V., Loriod B., Formisano-Tréziny C., Verrier L., Loundou A., Baumstarck-Barrau K., Boyer L., Gall V. (2012). Responder and Nonresponder Patients Exhibit Different Peripheral Transcriptional Signatures during Major Depressive Episode. Transl. Psychiatry.

[125] Fan H., Sun X., Guo W., Zhong A., Niu W., Zhao L., Dai Y., Guo Z., Zhang L., Lu J. (2014). Differential Expression of MicroRNA in Peripheral Blood Mononuclear Cells as Specific Biomarker for Major Depressive Disorder Patients. J. Psychiatr. Res..

[126] Hung Y.-Y., Wu M.-K., Tsai M.-C., Huang Y.-L., Kang H.-Y. (2019). Aberrant Expression of Intracellular Let-7e, MiR-146a, and MiR-155 Correlates with Severity of Depression in Patients with Major Depressive Disorder and Is Ameliorated after Antidepressant Treatment. Cells.

[127] Solich J., Kuśmider M., Faron-Górecka A., Pabian P., Kolasa M., Zemła B., Dziedzicka-Wasylewska M. (2020). Serum Level of MiR-1 and MiR-155 as Potential Biomarkers of Stress-Resilience of NET-KO and SWR/J Mice. Cells.

[128] Vaisvaser S., Modai S., Farberov L., Lin T., Sharon H., Gilam A., Volk N., Admon R., Edry L., Fruchter E. (2016). Neuro-Epigenetic Indications of Acute Stress Response in Humans: The Case of MicroRNA-29c. PLoS ONE.

[129] He S., Liu X., Jiang K., Peng D., Hong W., Fang Y., Qian Y., Yu S., Li H. (2016). Alterations of MicroRNA-124 Expression in Peripheral Blood Mononuclear Cells in Pre- and Post-Treatment Patients with Major Depressive Disorder. J. Psychiatr. Res..

[130] Sun N., Lei L., Wang Y., Yang C., Liu Z., Li X., Zhang K. (2016). Preliminary Comparison of Plasma Notch-Associated MicroRNA-34b and -34c Levels in Drug Naive, First Episode Depressed Patients and Healthy Controls. J. Affect. Disord..

[131] Saeedi S., Israel S., Nagy C., Turecki G. (2019). The Emerging Role of Exosomes in Mental Disorders. Transl. Psychiatry.

[132] Théry C., Witwer K.W., Aikawa E., Alcaraz M.J., Anderson J.D., Andriantsitohaina R., Antoniou A., Arab T., Archer F., Atkin-Smith G.K. (2018). Minimal Information for Studies of Extracellular Vesicles 2018 (MISEV2018): A Position Statement of the International Society for Extracellular Vesicles and Update of the MISEV2014 Guidelines. J. Extracell. Vesicles.

[133] Scioli M.G., Terriaca S., Fiorelli E., Storti G., Fabbri G., Cervelli V., Orlandi A. (2021). Extracellular Vesicles and Cancer Stem Cells in Tumor Progression: New Therapeutic Perspectives. Int. J. Mol. Sci..

[134] Malkin E.Z., Bratman S.V. (2020). Bioactive DNA from Extracellular Vesicles and Particles. Cell Death Dis..

[135] Hussain M.T., Iqbal A.J., Norling L.V. (2020). The Role and Impact of Extracellular Vesicles in the Modulation and Delivery of Cytokines during Autoimmunity. Int. J. Mol. Sci..

[136] Buzas E.I., György B., Nagy G., Falus A., Gay S. (2014). Emerging Role of Extracellular Vesicles in Inflammatory Diseases. Nat. Rev. Rheumatol..

[137] Hoshino A., Kim H.S., Bojmar L., Gyan K.E., Cioffi M., Hernandez J., Zambirinis C.P., Rodrigues G., Molina H., Heissel S. (2020). Extracellular Vesicle and Particle Biomarkers Define Multiple Human Cancers. Cell.

[138] Xu R., Rai A., Chen M., Suwakulsiri W., Greening D.W., Simpson R.J. (2018). Extracellular Vesicles in Cancer—Implications for Future Improvements in Cancer Care. Nat. Rev. Clin. Oncol..

[139] Goetzl E.J., Mustapic M., Kapogiannis D., Eitan E., Lobach I.V., Goetzl L., Schwartz J.B., Miller B.L. (2016). Cargo Proteins of Plasma Astrocyte-derived Exosomes in Alzheimer’s Disease. FASEB J..

[140] Shi M., Liu C., Cook T.J., Bullock K.M., Zhao Y., Ginghina C., Li Y., Aro P., Dator R., He C. (2014). Plasma Exosomal α-Synuclein Is Likely CNS-Derived and Increased in Parkinson’s Disease. Acta Neuropathol..

[141] Zhang Y., Zhao Y., Tian C., Wang J., Li W., Zhong C. (2018). Differential Exosomal MicroRNA Profile in the Serum of a Patient with Depression. Eur. J. Psychiatry.

[142] Liang J.-Q., Liao H.-R., Xu C.-X., Li X.-L., Wei Z.-X., Xie G.-J., Cheng Y. (2020). Serum Exosome-Derived MiR-139-5p as a Potential Biomarker for Major Depressive Disorder. Neuropsychiatr. Dis. Treat..

[143] Mizohata Y., Toda H., Koga M., Saito T., Fujita M., Kobayashi T., Hatakeyama S., Morimoto Y. (2021). Neural Extracellular Vesicle-Derived MiR-17 in Blood as a Potential Biomarker of Subthreshold Depression. Hum. Cell.

[144] Makeyev E.V., Zhang J., Carrasco M.A., Maniatis T. (2007). The MicroRNA MiR-124 Promotes Neuronal Differentiation by Triggering Brain-Specific Alternative Pre-MRNA Splicing. Mol. Cell.

[145] Yoo A.S., Sun A.X., Li L., Shcheglovitov A., Portmann T., Li Y., Lee-Messer C., Dolmetsch R.E., Tsien R.W., Crabtree G.R. (2011). MicroRNA-Mediated Conversion of Human Fibroblasts to Neurons. Nature.

[146] Nissan X., Blondel S., Navarro C., Maury Y., Denis C., Girard M., Martinat C., De Sandre-Giovannoli A., Levy N., Peschanski M. (2012). Unique Preservation of Neural Cells in Hutchinson- Gilford Progeria Syndrome Is Due to the Expression of the Neural-Specific MiR-9 MicroRNA. Cell Rep..

[147] Adlakha Y.K., Saini N. (2014). Brain MicroRNAs and Insights into Biological Functions and Therapeutic Potential of Brain Enriched MiRNA-128. Mol. Cancer.

[148] Enatescu V.R., Papava I., Enatescu I., Antonescu M., Anghel A., Seclaman E., Sirbu I.O., Marian C. (2016). Circulating Plasma Micro RNAs in Patients with Major Depressive Disorder Treated with Antidepressants: A Pilot Study. Psychiatry Investig..

[149] Su M., Hong J., Zhao Y., Liu S., Xue X. (2015). MeCP2 Controls Hippocampal Brain-Derived Neurotrophic Factor Expression via Homeostatic Interactions with MicroRNA-132 in Rats with Depression. Mol. Med. Rep..

[150] Chen Y., Shi J., Liu H., Wang Q., Chen X., Tang H., Yan R., Yao Z., Lu Q. (2020). Plasma MicroRNA Array Analysis Identifies Overexpressed MiR-19b-3p as a Biomarker of Bipolar Depression Distinguishing From Unipolar Depression. Front. Psychiatry.

[151] Marshe V.S., Islam F., Maciukiewicz M., Fiori L.M., Yerko V., Yang J., Turecki G., Foster J.A., Kennedy S.H., Blumberger D.M. (2020). Validation Study of MicroRNAs Previously Associated with Antidepressant Response in Older Adults Treated for Late-Life Depression with Venlafaxine. Prog. Neuropsychopharmacol. Biol. Psychiatry.

[152] Sun N., Yang C., He X., Liu Z., Liu S., Li X., Wang Y., Jin R., Zhang K. (2020). Impact of Expression and Genetic Variation of MicroRNA-34b/c on Cognitive Dysfunction in Patients with Major Depressive Disorder. Neuropsychiatr. Dis. Treat..

[153] Feng J., Wang M., Li M., Yang J., Jia J., Liu L., Zhou J., Zhang C., Wang X. (2019). Serum MiR-221-3p as a New Potential Biomarker for Depressed Mood in Perioperative Patients. Brain Res..

[154] Kim H.K., Tyryshkin K., Elmi N., Dharsee M., Evans K.R., Good J., Javadi M., McCormack S., Vaccarino A.L., Zhang X. (2019). Plasma MicroRNA Expression Levels and Their Targeted Pathways in Patients with Major Depressive Disorder Who Are Responsive to Duloxetine Treatment. J. Psychiatr. Res..

[155] Qi S., Yang X., Zhao L., Calhoun V.D., Perrone-Bizzozero N., Liu S., Jiang R., Jiang T., Sui J., Ma X. (2018). MicroRNA132 Associated Multimodal Neuroimaging Patterns in Unmedicated Major Depressive Disorder. Brain.

[156] Wang X., Wang B., Zhao J., Liu C., Qu X., Li Y. (2018). MiR-155 Is Involved in Major Depression Disorder and Antidepressant Treatment via Targeting SIRT1. Biosci. Rep..

[157] Volk N., Pape J.C., Engel M., Zannas A.S., Cattane N., Cattaneo A., Binder E.B., Chen A. (2016). Amygdalar MicroRNA-15a Is Essential for Coping with Chronic Stress. Cell Rep..

[158] Maffioletti E., Cattaneo A., Rosso G., Maina G., Maj C., Gennarelli M., Tardito D., Bocchio-Chiavetto L. (2016). Peripheral Whole Blood MicroRNA Alterations in Major Depression and Bipolar Disorder. J. Affect. Disord..

[159] Gururajan A., Naughton M.E., Scott K.A., O’Connor R.M., Moloney G., Clarke G., Dowling J., Walsh A., Ismail F., Shorten G. (2016). MicroRNAs as Biomarkers for Major Depression: A Role for Let-7b and Let-7c. Transl. Psychiatry.

[160] Liu Y., Yang X., Zhao L., Zhang J., Li T., Ma X. (2016). Increased MiR-132 Level Is Associated with Visual Memory Dysfunction in Patients with Depression. Neuropsychiatr. Dis. Treat..

[161] Camkurt M.A., Acar Ş., Coşkun S., Güneş M., Güneş S., Yılmaz M.F., Görür A., Tamer L. (2015). Comparison of Plasma MicroRNA Levels in Drug Naive, First Episode Depressed Patients and Healthy Controls. J. Psychiatr. Res..

[162] Cheng L.-C., Pastrana E., Tavazoie M., Doetsch F. (2009). MiR-124 Regulates Adult Neurogenesis in the Subventricular Zone Stem Cell Niche. Nat. Neurosci..

[163] Fischbach S.J., Carew T.J. (2009). MicroRNAs in Memory Processing. Neuron.

[164] Wang S.-S., Mu R.-H., Li C.-F., Dong S.-Q., Geng D., Liu Q., Yi L.-T. (2017). MicroRNA-124 Targets Glucocorticoid Receptor and Is Involved in Depression-like Behaviors. Prog. Neuropsychopharmacol. Biol. Psychiatry.

[165] Yang W., Liu M., Zhang Q., Zhang J., Chen J., Chen Q., Suo L. (2020). Knockdown of MiR-124 Reduces Depression-like Behavior by Targeting CREB1 and BDNF. Curr. Neurovasc. Res..

[166] Kozuka T., Omori Y., Watanabe S., Tarusawa E., Yamamoto H., Chaya T., Furuhashi M., Morita M., Sato T., Hirose S. (2019). MiR-124 Dosage Regulates Prefrontal Cortex Function by Dopaminergic Modulation. Sci. Rep..

[167] Mannironi C., Camon J., De Vito F., Biundo A., De Stefano M.E., Persiconi I., Bozzoni I., Fragapane P., Mele A., Presutti C. (2013). Acute Stress Alters Amygdala MicroRNA MiR-135a and MiR-124 Expression: Inferences for Corticosteroid Dependent Stress Response. PLoS ONE.

[168] Gao N., Tang H., Gao L., Tu G.-L., Luo H., Xia Y. (2020). LncRNA H19 Aggravates Cerebral Ischemia/Reperfusion Injury by Functioning as a CeRNA for MiR-19a-3p to Target PTEN. Neuroscience.

[169] Ge X.-L., Wang J.-L., Liu X., Zhang J., Liu C., Guo L. (2019). Inhibition of MiR-19a Protects Neurons against Ischemic Stroke through Modulating Glucose Metabolism and Neuronal Apoptosis. Cell. Mol. Biol. Lett..

[170] Mannironi C., Biundo A., Rajendran S., De Vito F., Saba L., Caioli S., Zona C., Ciotti T., Caristi S., Perlas E. (2018). MiR-135a Regulates Synaptic Transmission and Anxiety-Like Behavior in Amygdala. Mol. Neurobiol..

[171] Jauhari A., Singh T., Singh P., Parmar D., Yadav S. (2018). Regulation of MiR-34 Family in Neuronal Development. Mol. Neurobiol..

[172] Aranha M.M., Santos D.M., Solá S., Steer C.J., Rodrigues C.M.P. (2011). MiR-34a Regulates Mouse Neural Stem Cell Differentiation. PLoS ONE.

[173] De Antonellis P., Medaglia C., Cusanelli E., Andolfo I., Liguori L., De Vita G., Carotenuto M., Bello A., Formiggini F., Galeone A. (2011). MiR-34a Targeting of Notch Ligand Delta-Like 1 Impairs CD15+/CD133+ Tumor-Propagating Cells and Supports Neural Differentiation in Medulloblastoma. PLoS ONE.

[174] Lo Iacono L., Ielpo D., Parisi C., Napoli G., Accoto A., Di Segni M., Babicola L., D’Addario S.L., Guzzo S.M., Pascucci T. (2021). MicroRNA-34a Regulates 5-HT2C Expression in Dorsal Raphe and Contributes to the Anti-Depressant-like Effect of Fluoxetine. Neuropharmacology.

[175] Andolina D., Di Segni M., Bisicchia E., D’Alessandro F., Cestari V., Ventura A., Concepcion C., Puglisi-Allegra S., Ventura R. (2016). Effects of Lack of MicroRNA-34 on the Neural Circuitry Underlying the Stress Response and Anxiety. Neuropharmacology.

[176] Koshiol J., Wang E., Zhao Y., Marincola F., Landi M.T. (2010). Strengths and Limitations of Laboratory Procedures for MicroRNA Detection: Table 1. Cancer Epidemiol. Biomarkers Prev..

[177] Lee H.-M., Nguyen D.T., Lu L.-F. (2014). Progress and Challenge of MicroRNA Research in Immunity. Front. Genet..

